# Cell-Type-Specific Translation Profiling Reveals a Novel Strategy for Treating Fragile X Syndrome

**DOI:** 10.1016/j.neuron.2017.07.013

**Published:** 2017-08-02

**Authors:** Sophie R. Thomson, Sang S. Seo, Stephanie A. Barnes, Susana R. Louros, Melania Muscas, Owen Dando, Caoimhe Kirby, David J.A. Wyllie, Giles E. Hardingham, Peter C. Kind, Emily K. Osterweil

**Affiliations:** 1 Centre for Integrative Physiology/Patrick Wild Centre, University of Edinburgh, Hugh Robson Building, George Square, Edinburgh, EH8 9XD, UK; 2 Simons Initiative for the Developing Brain, University of Edinburgh, Hugh Robson Building, George Square, Edinburgh, EH8 9XD, UK; 3 UK Dementia Research Institute, University of Edinburgh, Hugh Robson Building, George Square, Edinburgh, EH8 9XD, UK

**Keywords:** fragile X, FMR1, autism, TRAP, protein synthesis, muscarinic receptor, m4, LTD, mglur theory, FMRP

## Abstract

Excessive mRNA translation downstream of group I metabotropic glutamate receptors (mGlu_1/5_) is a core pathophysiology of fragile X syndrome (FX); however, the differentially translating mRNAs that contribute to altered neural function are not known. We used translating ribosome affinity purification (TRAP) and RNA-seq to identify mistranslating mRNAs in CA1 pyramidal neurons of the FX mouse model (*Fmr1*^*−/y*^) hippocampus, which exhibit exaggerated mGlu_1/5_-induced long-term synaptic depression (LTD). In these neurons, we find that the *Chrm4* transcript encoding muscarinic acetylcholine receptor 4 (M_4_) is excessively translated, and synthesis of M_4_ downstream of mGlu_5_ activation is mimicked and occluded. Surprisingly, enhancement rather than inhibition of M_4_ activity normalizes core phenotypes in the *Fmr1*^*−/y*^, including excessive protein synthesis, exaggerated mGluR-LTD, and audiogenic seizures. These results suggest that not all excessively translated mRNAs in the *Fmr1*^*−/y*^ brain are detrimental, and some may be candidates for enhancement to correct pathological changes in the FX brain.

## Highlights

• TRAP-seq reveals altered translation of >120 mRNAs in *Fmr1*^*−/y*^ CA1 pyramidal neurons
• Muscarinic receptor M_4_ is excessively translated in *Fmr1*^*−/y*^ hippocampus
• Enhancement, not inhibition, of M_4_ corrects core phenotypes in the *Fmr1*^*−/y*^ mouse
• Not all excessively translating mRNAs are detrimental to *Fmr1*^*−/y*^ brain function

## Teaser

Thomson et al. use TRAP-seq to identify excessive translation of muscarinic receptor M_4_ in *Fmr1*^*−/y*^ neurons. Surprisingly, enhancement rather than inhibition of M_4_ corrects neurological *Fmr1*^*−/y*^ phenotypes. This suggests that elevated translation of certain mRNAs in the FX brain may be protective rather than pathological.

## Introduction

Several genetic mutations that affect neuronal protein synthesis have been linked to the development of autism and intellectual disability (ASD/ID) (Kelleher and Bear, 2008, Louros and Osterweil, 2016). Fragile X syndrome (FX), a prominent single-gene cause of ASD/ID, arises from mutations in the *FMR1* gene that encodes the protein synthesis repressor fragile X mental retardation protein (FMRP) (Ashley et al., 1993). In hippocampal CA1, FMRP is synthesized at synapses by activation of group I metabotropic glutamate receptors (mGlu_1/5_), where it acts as a negative regulator of the mRNA translation supporting long-term synaptic depression (LTD) (Bear et al., 2004, Weiler et al., 1997). In the FX mouse model (*Fmr1*^*−/y*^), loss of FMRP leads to excessive protein synthesis downstream of mGlu_1/5_ activation, and consequently, mGluR-LTD is exaggerated and no longer dependent upon new protein synthesis (Huber et al., 2002, Nosyreva and Huber, 2006).

According to the mGluR theory of fragile X, excessive translation underlies several neurological pathologies in FX, and numerous studies support this interpretation (Bear et al., 2004, Stoppel et al., 2017). Excessive protein synthesis has been observed in multiple brain regions of the *Fmr1*^*−/y*^ mouse (Dölen et al., 2007, Osterweil et al., 2010, Qin et al., 2005), and several strategies that reduce protein synthesis have been shown to correct pathological phenotypes (Bhattacharya et al., 2016, Gross et al., 2015, Henderson et al., 2012, Liu et al., 2012, Michalon et al., 2012, Osterweil et al., 2013). Although there have been excellent studies identifying FMRP target mRNAs (Brown et al., 2001, Darnell et al., 2011), as well as proteins differentially expressed in the *Fmr1*^*−/y*^ brain (Klemmer et al., 2011, Liao et al., 2008, Tang et al., 2015), there is little known about the identities of the mistranslating mRNAs that contribute to neurological deficits in FX. If aberrant mRNA translation is indeed a core pathophysiology, then the challenge becomes isolating and interpreting the changes in translation that result in altered function.

In this study, we employed a combination of cell-type-specific translating ribosome affinity purification (TRAP) and RNA sequencing (RNA-seq) to identify differentially translating mRNAs in CA1 pyramidal neurons of the *Fmr1*^*−/y*^ hippocampus (Heiman et al., 2008). We focused on CA1 pyramidal neurons based on work showing that excessive translation in these neurons leads to functional disruption, namely the exaggeration of mGluR-LTD in the *Fmr1*^*−/y*^ mouse (Nosyreva and Huber, 2006). This first cell-type-specific translation analysis identified 121 differentially translating mRNAs in *Fmr1*^*−/y*^ CA1 neurons. Interestingly, the muscarinic acetylcholine receptor (mAChR) signaling pathway is the most significantly changed gene category, with the *Chrm4* mRNA encoding muscarinic subtype M_4_ significantly overexpressed in the *Fmr1*^*−/y*^. Further experiments confirmed the over-translation of *Chrm4* and subsequent overexpression of M_4_ in *Fmr1*^*−/y*^ hippocampus. Based on these results, we examined whether inhibition of M_4_ could correct pathological changes in the *Fmr1*^*−/y*^ brain. To our surprise, we find that the opposite strategy, an enhancement of M_4_ using the highly specific positive allosteric modulator (PAM) VU0152100, normalizes excessive protein synthesis and exaggerated mGluR-LTD in the *Fmr1*^*−/y*^ hippocampus. Furthermore, systemic injection of VU0152100 significantly reduces the incidence of audiogenic seizures (AGS) in *Fmr1*^*−/y*^ mice. These results suggest that not all excessively translated mRNAs in the *Fmr1*^*−/y*^ brain are contributing to pathological changes. Instead, one of the most significantly over-translated mRNAs in *Fmr1*^*−/y*^ CA1 neurons encodes a protein that should be positively modulated rather than inhibited to correct brain function.

## Results

### Isolation of Translating mRNAs from Hippocampal CA1 Pyramidal Neurons Using TRAP

**Figure 1:**
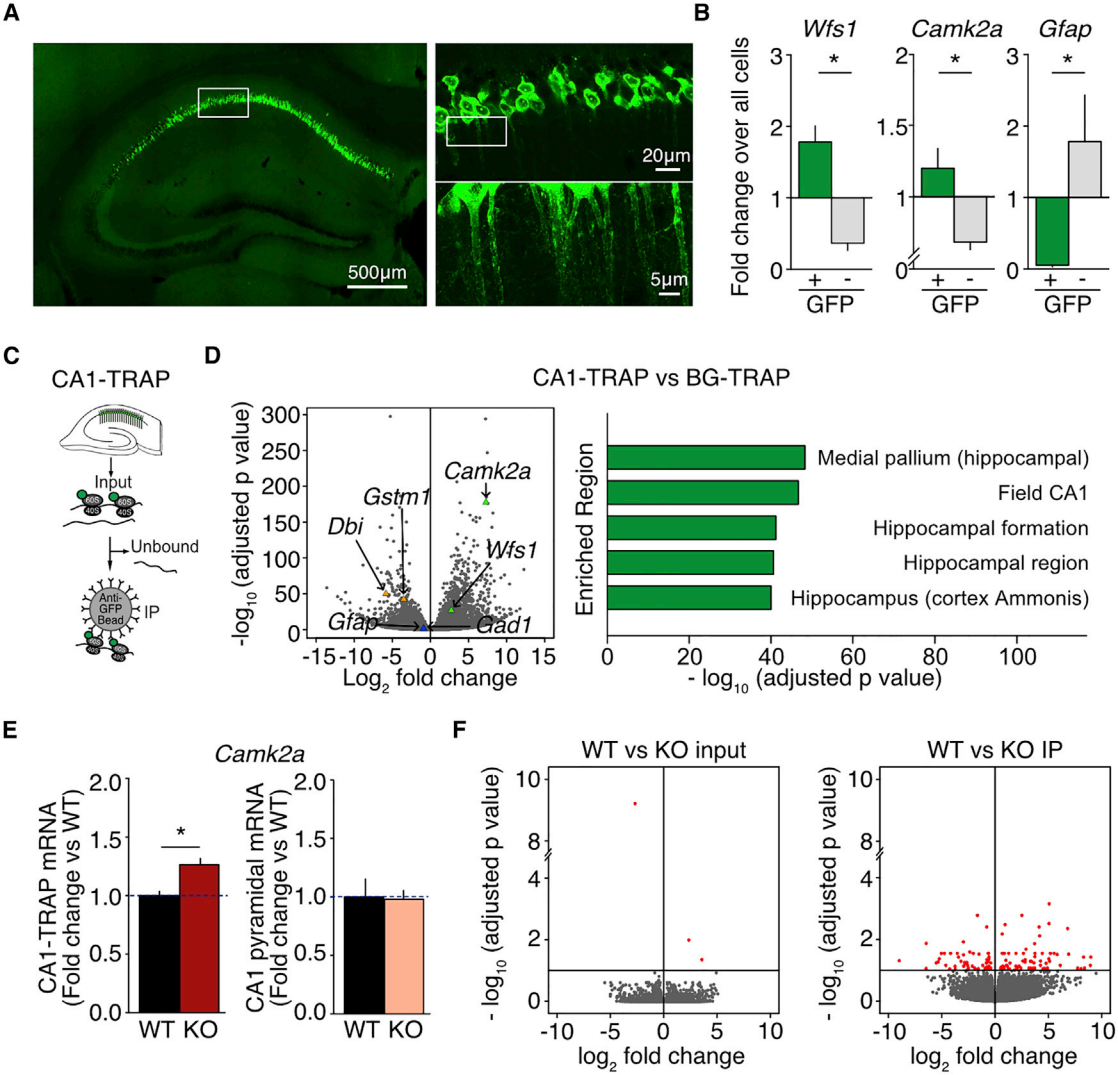
TRAP-Seq Identifies Differentially Translating mRNAs in *Fmr1*^*−/y*^ CA1 Pyramidal Neurons (A) Confocal images show selective expression of GFP-L10a in pyramidal neurons of the CA1 region. (B) GFP-positive (GFP+) cells in CA1-TRAP hippocampus are enriched for CA1 neuronal markers (*Camk2a*: GFP− 0.683 ± 0.05, GFP+ 1.200 ± 0.138, ^∗^p = 0.0046, n = 12; *Wfs1*: GFP− 0.370 ± 0.104, GFP+ 1.781 ± 0.224, ^∗^p < 0.0001, n = 9) and depleted of glial markers (*Gfap*: GFP− 1.784 ± 0.650, GFP+ 0.054 ± 0.022, ^∗^p = 0.0218, n = 12) compared to all cells. (C) Schematic representation of TRAP shows isolation of translating ribosomes (IP) from Input using anti-GFP coated beads. (D) Differentially expressed genes in CA1-TRAP versus Bergmann glia (BG)-specific TRAP are enriched in CA1 neuronal markers according to the Allen Brain Atlas enrichment algorithm. (E) *Camk2a* is significantly increased in *Fmr1*^*−/y*^ versus WT CA1-TRAP IP (WT = 1.00 ± 0.037, KO = 1.26 ± 0.054, ^∗^p = 0.0004, n = 14). Total *Camk2a* is equivalent in *Fmr1*^*−/y*^ and WT FACs-isolated CA1 pyramidal neurons (WT = 1.00 ± 0.059, KO = 0.855 ± 0.047, p = 0.0734, n = 9). (F) TRAP-seq analysis reveals differential expression of 121 genes in the IP fraction and 3 genes in the Input fraction (FDR < 0.1). n = number of littermate pairs. Error bars indicate SEM.

In *Fmr1*^*−/y*^ CA1, excessive translation contributes to the exaggeration of mGluR-LTD (Huber et al., 2002). To isolate differentially translating mRNAs specifically from CA1 pyramidal neurons, we used a TRAP strategy that allows for cell-type-specific isolation of translating mRNAs using bacterial artificial chromosome (BAC) transgenic mouse lines engineered to express a GFP-tagged L10a ribosomal subunit in select cell populations (Heiman et al., 2008). The association of the L10a subunit with the 60S large ribosomal subunit allows for the enrichment of translating mRNAs (Heiman et al., 2008, Katz et al., 2016). For our study, we used a BAC transgenic line that shows a CA1 pyramidal-specific expression of GFP-L10a within the hippocampus (referred to as CA1-TRAP) (Doyle et al., 2008, Tao et al., 2016). Confocal imaging of coronal brain sections from this CA1-TRAP mouse confirms an expression of GFP-L10a within both the soma and dendrites of pyramidal neurons in the CA1 region (Figure 1A). Analysis of GFP-expressing (GFP+) cells isolated by fluorescence-activated cell sorting (FACS) reveals an enrichment of the CA1 pyramidal neuron marker *Wfs1* (^∗^p < 0.0001) and the excitatory neuron marker *Camk2a* (^∗^p = 0.0046) as compared to total hippocampal cells. In contrast, the glial marker *Gfap* is depleted (^∗^p = 0.0218; Figure 1B). This confirms that the GFP-L10a-expressing cells are indeed CA1 pyramidal neurons. To ensure that we could isolate CA1-specific translating mRNAs, we performed TRAP immunoprecipitations (IPs) from hippocampi isolated from CA1-TRAP mice using previously established protocols (Heiman et al., 2008) (Figure 1C). Ribosome-bound transcripts were analyzed using RNA-seq, and the identified genes were compared to previously published datasets from cerebellar Bergmann glia (BG), Purkinje cells (PCs), and granule cells (GCs) (Mellén et al., 2012). The results of these comparisons show a significant enrichment of CA1 pyramidal neuron markers in the translating ribosome fraction (Figure 1D; Figure S1). These results confirm that mRNAs isolated in the CA1-TRAP IP originate from CA1 pyramidal neurons.

### RNA-Seq Identifies Differentially Translating mRNAs in *Fmr1*^*−/y*^ CA1 Pyramidal Neurons

To identify differentially translating mRNAs in *Fmr1*^*−/y*^ CA1 neurons, we compared *Fmr1*^*−/y*^ (knockout [KO]) and wild-type (WT) littermate mice, each heterozygous for the CA1-TRAP transgene. All experiments were performed with the experimenter blind to genotype. In order to confirm that changes seen in *Fmr1*^*−/y*^ TRAP-bound mRNAs are consistent with changes seen using other methodologies, we measured the expression of *Camk2a*, previously shown to be over-translated in multiple studies (Darnell et al., 2011, Osterweil et al., 2010, Zalfa et al., 2003). Our results show a significant enrichment of *Camk2a* in the *Fmr1*^*−/y*^ CA1-TRAP fraction (^∗^p = 0.0004) (Figure 1E). In contrast, total *Camk2a* expression is not elevated in FACS-isolated CA1 pyramidal neurons from *Fmr1*^*−/y*^ hippocampus, suggesting that changes seen in the TRAP fraction are not due to changes in the transcription of *Camk2a* (Figure 1E).

After verifying that the *Fmr1*^*−/y*^ CA1-TRAP reflects previously reported changes in translation, we performed RNA-seq on six sets of *Fmr1*^*−/y*^ and WT CA1-TRAP littermates (see STAR Methods). Hippocampi were isolated from *Fmr1*^*−/y*^ and WT littermates at a juvenile age (postnatal days 25–32) when the exaggerated mGluR-LTD phenotype is observed (Nosyreva and Huber, 2006). RNA was isolated from both the TRAP fraction and the starting Input, and samples were processed for RNA-seq according to established protocols (see STAR Methods). Differential gene expression was determined using DESeq2 at the default false discovery rate (FDR) of 0.1, consistent with previous studies (Cho et al., 2015, Tao et al., 2016). Our results show that 121 genes are differentially expressed in the *Fmr1*^*−/y*^ CA1-TRAP fraction (Figure 1F). The majority of differentially translating transcripts are increased in the *Fmr1*^*−/y*^ versus WT (Table S1); however, a significant number are also decreased (Table S2). In contrast to the ribosome-bound TRAP fraction, a comparison of WT and *Fmr1*^*−/y*^ Input fractions reveals only three differentially expressed genes (Figure 1F). This is consistent with the observed increase in translation, but not transcription, seen in the *Fmr1*^*−/y*^ hippocampus (Muddashetty et al., 2007, Osterweil et al., 2010).

### FMRP Target mRNAs Are Downregulated in *Fmr1*^*−/y*^ Hippocampus

**Figure 2:**
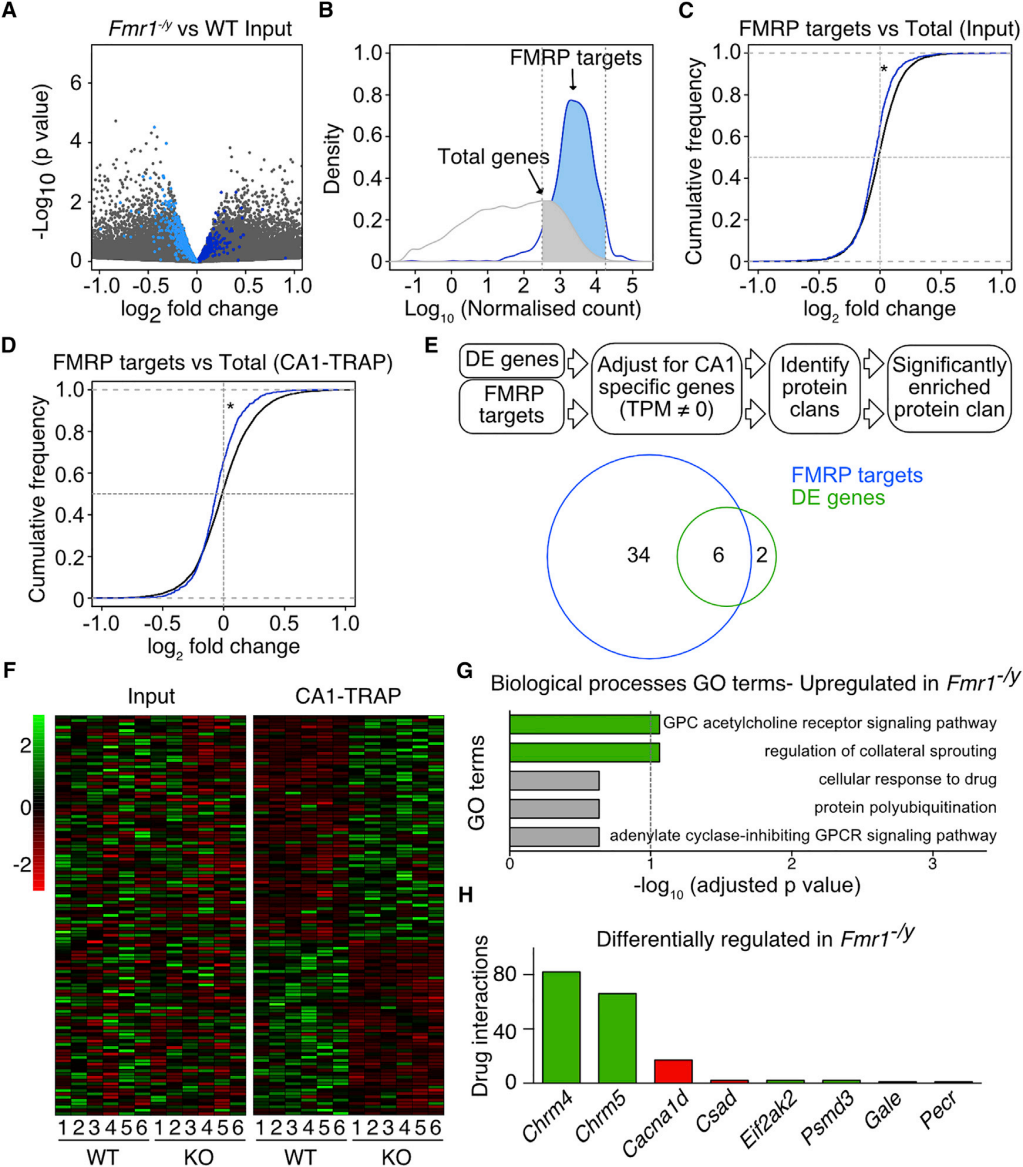
Differentially Translated mRNAs in *Fmr1*^*−/y*^ CA1 Include FMRP Targets and mAChR Transcripts (A) Differential expression analysis shows gene changes in WT versus *Fmr1*^*−/y*^ Input fraction, with FMRP targets highlighted in blue. (B) FMRP targets were compared to differentially expressed (total) genes with the same level of abundance (normalized count between 10^2.5^ and 10^4.25^). (C and D) A cumulative distribution of FMRP targets shows a significant shift toward downregulation when compared to the distribution of total differentially expressed genes with the same level of abundance in both Input (C) and CA1-TRAP (D) fractions (K-S test, p = 9.23 × 10^−14^, p = 4.86 × 10^−13^). (E) Pfam analysis of enriched protein families reveals that six out of eight pClans enriched in the differentially expressed (DE) *Fmr1*^*−/y*^ CA1-TRAP gene list overlap with pClans enriched in the CA1-adjusted FMRP target list. (F) Heatmap shows the fold change of differentially expressed genes in each pair of *Fmr1*^*−/y*^ versus WT (IP and Input fractions). (G) GO analysis identifies G-protein-coupled acetylcholine receptor signaling pathway as the most enriched functional category in the upregulated *Fmr1*^*−/y*^ CA1-TRAP gene list. (H) Drug gene interaction database reveals that *Chrm4* is the most amenable target pharmacologically. Upregulated genes are highlighted in green, and downregulated genes are highlighted in red. n = number of littermate pairs. Error bars indicate SEM.

The number of mRNA targets of FMRP is estimated to be well over 800, and it is believed that many of these are translationally repressed when bound to FMRP (Darnell et al., 2011). However, it is not clear how many of these mRNAs are over-translating in the *Fmr1*^*−/y*^ brain. Our analysis of differentially translating mRNAs identified only three verified FMRP targets (*Cacna1d*, *Arhgef17*, and *Pcdhgc5*), and all are downregulated in the *Fmr1*^*−/y*^ TRAP (Table S2) (Darnell et al., 2011). This surprising result motivated us to investigate the global expression difference in all FMRP target mRNAs in both the Input (i.e., total hippocampal mRNA) and CA1-TRAP fractions of the *Fmr1*^*−/y*^ hippocampus as compared to WT (Figure 2A). To do this, we compared the differential expression of FMRP target mRNAs to all genes expressed at the same level of abundance (Figure 2B). Interestingly, a cumulative distribution of FMRP targets in the differentially expressed population shows a significant shift toward downregulation (Kolmogorov-Smirnov [K-S] test ^∗^p = 9.23 × 10^−14^) (Figure 2C). The same significant shift was seen when the FMRP target list was compared to five randomly generated gene sets of the same size (Figure S2). This indicates a subtle reduction in the expression of FMRP targets in the *Fmr1*^*−/y*^ hippocampal mRNA population when compared to WT. To examine whether the reduction in FMRP target expression was reflected in the translating ribosome fraction, we repeated this analysis using CA1-TRAP samples. The results show the same difference in the distribution of FMRP targets versus WT (K-S test ^∗^p = 4.86 × 10^−13^) (Figure 2D; Figure S2). Thus, the reduced expression of FMRP targets in the total *Fmr1*^*−/y*^ mRNA Input is mirrored in the CA1-TRAP fraction. The conclusion from our analysis is that FMRP target mRNAs are not necessarily enriched in the translating ribosome fraction of the *Fmr1*^*−/y*^ hippocampus at this age.

One possibility suggested by our findings is that differentially translating mRNAs in the juvenile *Fmr1*^*−/y*^ hippocampus are not necessarily reflective of a proximal loss of FMRP, but rather they represent a homeostatic shift that has developed in response to an early loss of FMRP. We thus wondered whether the differentially translating mRNAs that we identified were compensating for the downregulated FMRP targets. To investigate this, we examined whether the differentially expressed transcripts in the *Fmr1*^*−/y*^ CA1-TRAP encoded proteins similar to those encoded by FMRP targets. To sort these transcripts by function, we used a Pfam analysis to categorize differentially expressed mRNAs and FMRP targets into protein clans (pClans) (Finn et al., 2016). The enrichment of the pClans in each list was determined using a background list of CA1 specific genes (see STAR Methods). Our analysis revealed that of the eight pClans enriched in the list of differentially expressed transcripts, six of them were enriched in the FMRP target list (Figure 2E; Table S3), indicating that the majority of differentially translating mRNAs in *Fmr1*^*−/y*^ CA1 neurons are functionally similar to FMRP targets. It is possible that these changes are compensatory adaptations to the original loss of FMRP and subsequent dysregulation of FMRP target mRNAs.

### Muscarinic Acetylcholine Receptors Are Excessively Translated in *Fmr1*^*−/y*^ CA1 Pyramidal Neurons

A major aim of our TRAP-seq experiments was to identify the excessively translating mRNAs in *Fmr1*^*−/y*^ CA1 that underlie altered function. We began by performing an unbiased gene ontology (GO) analysis to determine whether any particular gene category was enriched in the differentially translating population isolated from the six sets of *Fmr1*^*−/y*^ CA1-TRAP hippocampi. Separate analyses were performed for all differentially expressed transcripts, upregulated transcripts, and downregulated transcripts in the *Fmr1*^*−/y*^ CA1-TRAP (Figure 2F; Tables S4–S6). Interestingly, our analysis of both total transcripts and upregulated transcripts revealed a significant enrichment of the G-protein-coupled (GPC) acetylcholine receptor (mAChR) signaling pathway (Figure 2G; Tables S4 and S5). Specifically, the *Chrm4* and *Chrm5* genes encoding the M_4_ and M_5_ receptors were upregulated in the *Fmr1*^*−/y*^ CA1-TRAP samples (Tables S4 and S5). This result was particularly interesting in light of the well-known role of mAChRs, including M_4_, in the modulation of synaptic plasticity and excitability in the hippocampus (Bubser et al., 2012). In addition to this analysis, we also wondered whether any of the differentially expressed mRNAs in the *Fmr1*^*−/y*^ CA1-TRAP encoded targets for pharmacological intervention. We investigated this using the recently developed Drug Gene Interaction database (DGIdb; http://dgidb.genome.wustl.edu/), which ranks gene sets based on number of known drug interactions (Griffith et al., 2013). The results identified *Chrm4* as the top candidate in our list (Figure 2H). Thus, the muscarinic receptor family represented both the most significantly overexpressed gene category in the *Fmr1*^*−/y*^ CA1-TRAP and the most amenable to pharmacological manipulation.

**Figure 3:**
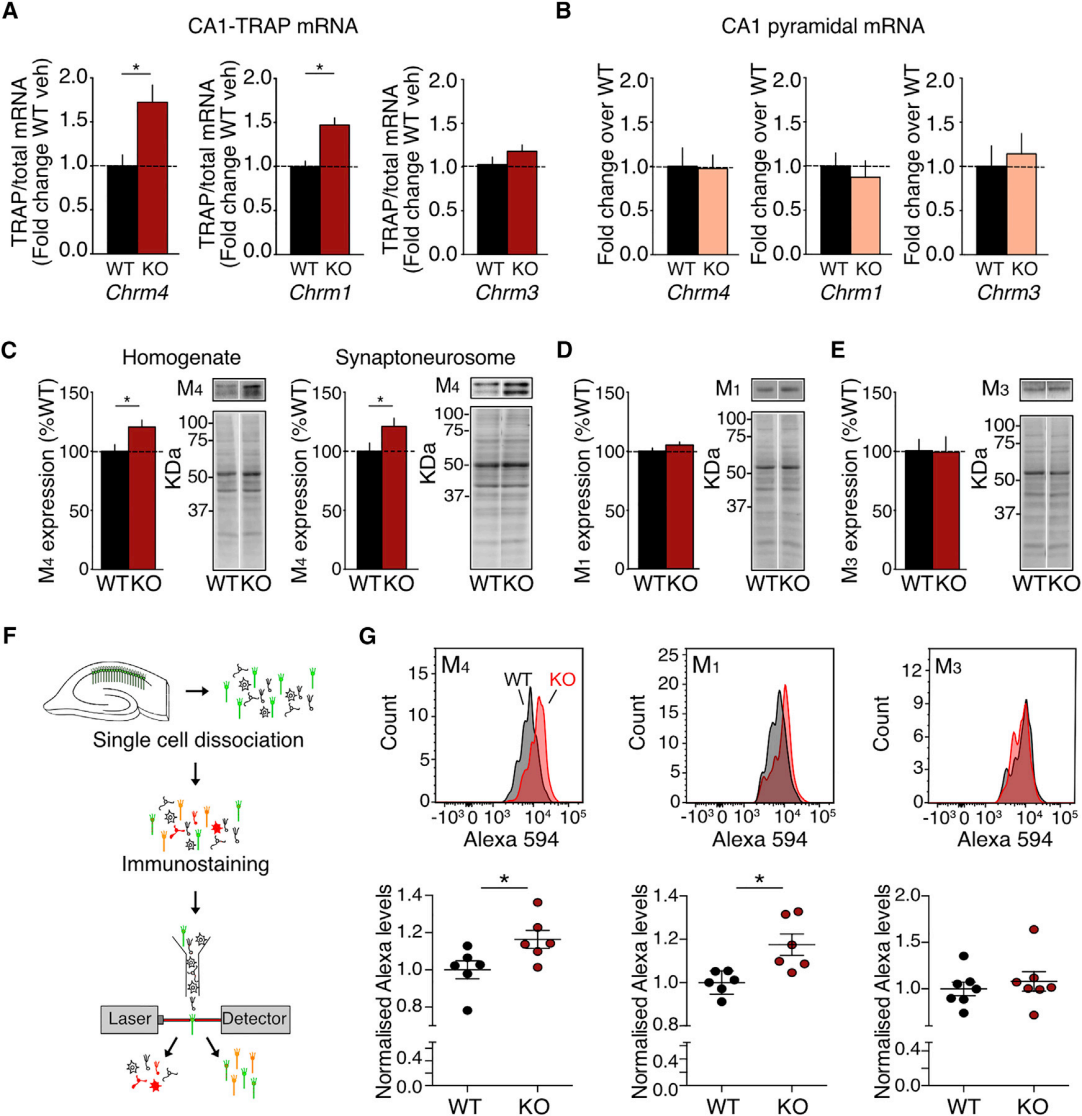
M_1_ and M_4_ Are Excessively Synthesized and Overexpressed in *Fmr1*^*−/y*^ CA1 Pyramidal Neurons (A) *Chrm4* and *Chrm1*, but not *Chrm3*, are enriched in the *Fmr1*^*−/y*^ CA1-TRAP IP (*Chrm4*: WT = 1.00 ± 0.124, KO = 1.72 ± 0.195, ^∗^p = 0.0044, n = 14; *Chrm1*: WT = 1.00 ± 0.062, KO = 1.47 ± 0.079, ^∗^p < 0.0001, n = 14; *Chrm3*: WT = 1.00 ± 0.085, KO = 1.184 ± 0.073, p = 0.192, n = 10). (B) Total mRNA levels of *Chrm4, Chrm1* and *Chrm3* are unchanged in FACS-isolated *Fmr1*^*−/y*^ CA1 pyramidal neurons (*Chrm4*: WT = 1.00 ± 0.209, KO = 0.98 ± 0.154, p = 0.926, n = 9; *Chrm1*: WT = 1.00 ± 0.150, KO = 0.87 ± 0.185, p = 0.602, n = 11; *Chrm3*: WT = 1.00 ± 0.232, KO = 1.14 ± 0.230, p = 0.668, n = 8). (C) Immunoblotting shows overexpression of M_4_ protein in hippocampal slice homogenates (WT = 100% ± 5.7%, KO = 121% ± 5.7%, ^∗^p = 0.0186, n = 12) and synaptoneurosomes (WT = 100% ± 7.10%, KO = 121% ± 6.89%, ^∗^p = 0.0203, n = 9). (D and E) Hippocampal slice homogenates show no difference in M_1_ (D) (WT = 100% ± 2.5%, KO = 105% ± 2.5%, p = 0.129, n = 12) or M_3_ (E) (WT = 100% ± 9.5%, KO = 98% ± 12.9%, p = 0.94, n = 9) expression. (F) Schematic shows steps for FACS immunostaining. (G) FACS-immunostaining reveals significant increase in the expression of M_4_ and M_1_ (M_4_: WT = 0.925 ± 0.045, KO = 1.075 ± 0.045, ^∗^p = 0.0389, n = 6; M_1_: WT = 0.920 ± 0.002, KO = 1.080 ± 0.046, ^∗^p = 0.0092, n = 6), but not M_3_ (WT = 0.962 ± 0.07, KO = 1.038 ± 0.1011 p = 0.547, n = 7), in *Fmr1*^*−/y*^ CA1 pyramidal neurons. n = number of littermate pairs. Error bars indicate SEM.

The mAChR family is comprised of five subtypes, which are coupled to either the G_q_-PLC (M_1_, M_3_, and M_5_) or G_i/o_/G_s_-cAMP (M_2_ and M_4_) signaling pathways (Kruse et al., 2014). Of these subtypes, M_1_, M_4_, and M_3_ are the most predominantly expressed in the hippocampus (Zang and Creese, 1997). To assess the translation of these receptors in *Fmr1*^*−/y*^ versus WT CA1, we performed additional TRAP experiments and measured the levels of *Chrm4*, as well as of *Chrm1* and *Chrm3*, using quantitative PCR (qPCR). Our results revealed a significant overexpression of *Chrm4* (^∗^p = 0.0044) and *Chrm1* (^∗^p < 0.0001), but not *Chrm3*, in the *Fmr1*^*−/y*^ CA1-TRAP IP (Figure 3A). Although we also validated the increased expression of *Chrm5* in the *Fmr1*^*−/y*^ CA1-TRAP (^∗^p = 0.041), this transcript is much less abundant in the hippocampus (Figure S3). We therefore focused our further analyses on the M_1_, M_3_, and M_4_ subtypes.

To determine whether the increased expression of *Chrm4* and *Chrm1* in the *Fmr1*^*−/y*^ TRAP was due to a change in the basal expression of these transcripts in CA1 pyramidal neurons, we examined total mRNA expression in FACS-isolated GFP-L10a-expressing neurons. qPCR analyses revealed no elevation in *Chrm4*, *Chrm1*, or *Chrm3* transcripts in these cells, suggesting that the increased expression of these transcripts in the translating ribosome fraction is not driven by an underlying change in transcription (Figure 3B). Next, we investigated whether the increased translation of *Chrm4* and *Chrm1* resulted in an increased expression of M_4_ and M_1_ receptors in the *Fmr1*^*−/y*^ hippocampus. Consistent with our previous experiments using TRAP, quantitative immunoblotting of hippocampal slice homogenate shows a significant increase in M_4_ expression in the *Fmr1*^*−/y*^ hippocampus (^∗^p = 0.0186; Figure 3C). This increase is also seen in synaptoneurosome fractions isolated from *Fmr1*^*−/y*^ hippocampus (^∗^p = 0.0203), suggesting that M_4_ is overexpressed at the synapse. Similar to the CA1-TRAP results, no change was observed in the expression of M_3_ in *Fmr1*^*−/y*^ hippocampal homogenates (Figure 3D). Interestingly, the expression of M_1_ was also not significantly changed in *Fmr1*^*−/y*^ hippocampal homogenate (Figure 3E) despite the increase in *Chrm1* observed in *Fmr1*^*−/y*^ CA1-TRAP. These results suggested that either the increased translation of *Chrm1* did not result in M_1_ overexpression or the increase in M_1_ was occluded by the presence of other cell types in a whole hippocampal homogenate. To distinguish between these possibilities, we used a combination of FACS and immunostaining to measure the levels of M_4_, M_1_, and M_3_ selectively in CA1 pyramidal neurons isolated from the hippocampus. Neurons from CA1-TRAP WT and *Fmr1*^*−/y*^ hippocampi were dissociated and immunostained for M_4_, M_1_, or M_3_ using Alexa 594-conjugated secondary antibodies, and the expression levels for all three receptors in the GFP+ cell population were determined by quantitative fluorescence measurements (see STAR Methods; Figure 3F). Supporting our TRAP results, we find a significant increase in the expression of M_4_ and M_1_, but not M_3_, in CA1 neurons isolated from the *Fmr1*^*−/y*^ hippocampus (M_4_^∗^p = 0.0389, M_1_^∗^p = 0.0092, M_3_ p = 0.547; Figure 3G). These results confirm that the over-translation of *Chrm4* and *Chrm1* leads to an overexpression of M_1_ and M_4_ receptors in *Fmr1*^*−/y*^ CA1 pyramidal neurons.

### M_4_ Is Translated Downstream of mGlu_5_ Activation

**Figure 4:**
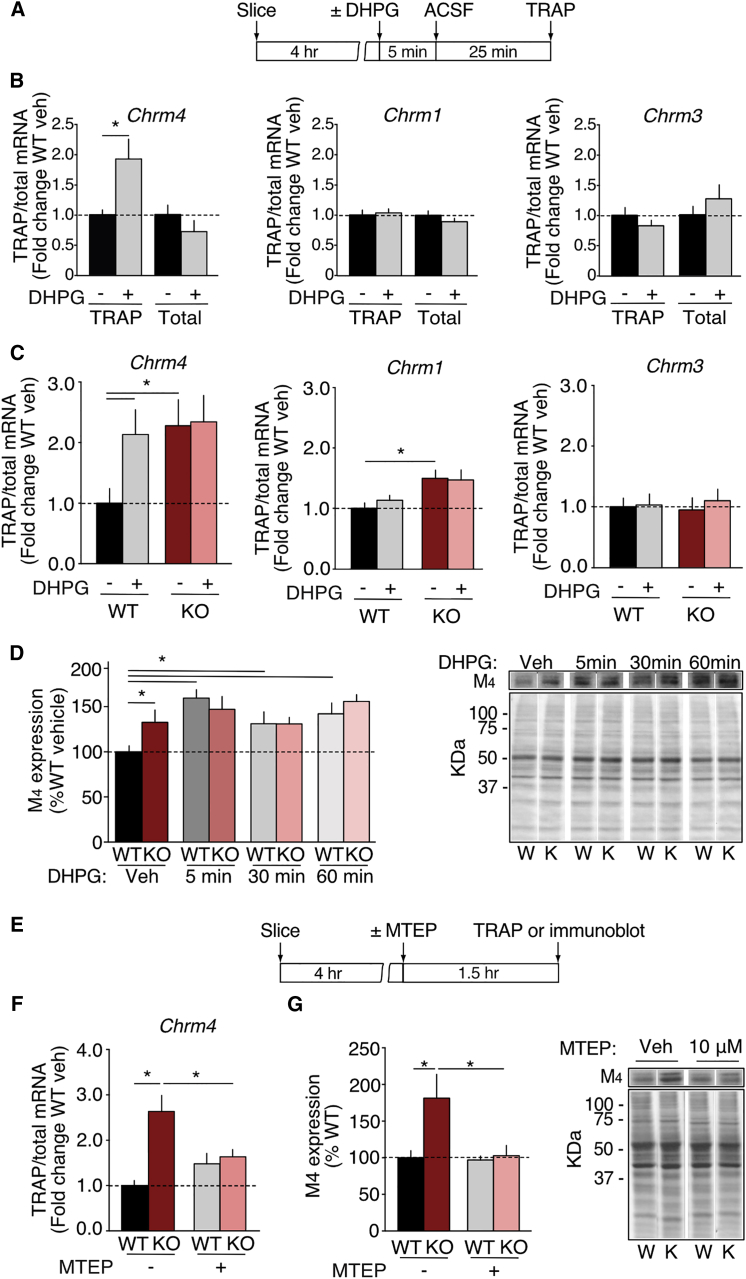
M_4_ Synthesis Downstream of mGlu_5_ Is Mimicked and Occluded in the *Fmr1*^*−/y*^ Hippocampus (A) Time course for DHPG stimulation experiments. (B) Analysis of transcripts encoding hippocampal mAChR subunits reveals a striking upregulation of *Chrm4* mRNA in CA1-TRAP IP after mGlu_1/5_ stimulation (Veh = 1.00 ± 0.12, DHPG = 1.72 ± 0.23, ^∗^p = 0.0047, n = 15), with no changes seen in *Chrm1* or *Chrm3* (*Chrm1*: Veh = 1.00 ± 0.07, DHPG = 1.03 ± 0.06, p = 0.75, n = 16; *Chrm3*: Veh = 1.00 ± 0.12, DHPG = 0.82 ± 0.09, p = 0.209, n = 14). (C) DHPG stimulation of WT slices shows dramatic increase in *Chrm4* mRNA in the TRAP IP fraction. In *Fmr1*^*−/y*^ slices, *Chrm4* mRNA is already significantly elevated in the TRAP IP and does not increase further with mGlu_1/5_ activation (WT vehicle = 1.00 ± 0.24, WT DHPG = 2.13 ± 0.41, KO vehicle = 2.28 ± 0.43, KO DHPG = 2.34 ± 0.44, ANOVA genotype ^∗^p = 0.03, treatment ^∗^p = 0.048, WT versus KO veh ^∗^p = 0.03, KO veh versus DHPG p > 0.999, n = 7). *Chrm1* mRNA is significantly elevated in the *Fmr1*^*−/y*^ CA1-TRAP IP, but DHPG does not increase *Chrm1* in either WT or *Fmr1*^*−/y*^ CA1-TRAP IPs (WT vehicle = 1.00 ± 0.09, WT DHPG = 1.13 ± 0.08, KO vehicle = 1.50 ± 0.13, KO DHPG = 1.47 ± 0.16, ANOVA genotype ^∗^p = 0.005, treatment p = 0.753, WT versus KO veh ^∗^p = 0.04, WT veh versus DHPG p = 0.944, n = 7). *Chrm3* mRNA is neither increased in the *Fmr1*^*−/y*^ CA1-TRAP IP nor elevated with DHPG (WT veh = 1.00 ± 0.14, WT DHPG, = 1.029 ± 0.18, KO veh = 0.946 ± 0.19, KO DHPG = 1.09 ± 0.19, ANOVA genotype p = 0.97, treatment p = 0.55, n = 7). (D) Immunoblotting shows a robust increase in M_4_ expression in WT slices after 5 min of DHPG stimulation, which is maintained at 30 min and 60 min post-stimulation. In contrast, the elevated expression of M_4_ in *Fmr1*^*−/y*^ slices is not further increased with DHPG stimulation (WT vehicle = 100% ± 6.63%, WT DHPG 5 min = 159.59% ± 9.37%, WT DHPG 30 min = 131.09% ± 13.23%, WT DHPG 60 min = 141.66% ± 12.08%, KO vehicle = 132.70% ± 7.31%, KO DHPG 5 min = 146.95% ± 7.94% KO DHPG 30 min = 131.04% ± 13.01%, KO DHPG 60 min = 155.75% ± 10.28%, ANOVA treatment × genotype ^∗^p = 0.037, n = 7). (E) Time course for MTEP slice experiments. (F) Incubation with 10 μM MTEP reduces *Chrm4* in the *Fmr1*^*−/y*^ CA1-TRAP to WT levels (WT vehicle = 1.00 ± 0.112, WT MTEP = 1.48 ± 0.230, KO vehicle = 2.63 ± 0.352, KO MTEP = 1.63 ± 0.161, ANOVA genotype ^∗^p = 0.0014, treatment p = 0.1923, genotype × treatment ^∗^p = 0.0119, WT veh versus KO veh ^∗^p = 0.0024, WT veh versus WT MTEP p = 0.306, KO veh versus KO MTEP ^∗^p = 0.0289, WT MTEP versus KO MTEP p = 0.880, n = 8). (G) Immunoblotting shows a significant reduction in M_4_ expression in MTEP-treated *Fmr1*^*−/y*^ slices (WT vehicle = 100% ± 9.7%, WT MTEP = 97% ± 5.4%, KO vehicle = 181% ± 32.6%, KO MTEP = 103% ± 14.1%, ANOVA genotype ^∗^p = 0.034, WT veh versus KO veh ^∗^p = 0.0181, KO veh versus KO MTEP ^∗^p = 0.0145, n = 6). n = number of littermate pairs. Error bars indicate SEM.

Protein synthesis downstream of mGlu_1/5_ is elevated in the *Fmr1*^*−/y*^ brain, and this occludes further translation (Bassell and Warren, 2008, Osterweil et al., 2010). We thus wondered whether the translation of *Chrm4* and *Chrm1* was stimulated by mGlu_1/5_ activation and whether this is saturated in the *Fmr1*^*−/y*^ hippocampus. To test this, we prepared hippocampal slices from WT CA1-TRAP mice and stimulated them with 50 μM of the mGlu_1/5_ agonist S-3,5-Dihydroxyphenylglycine (S-DHPG) in a manner that induces mGluR-LTD (Figure 4A). To ensure that our assay accurately reflected mGlu_1/5_-stimulated translation, we quantified the levels of mRNA encoding the cytoskeletal plasticity protein Arc, which is translated in response to DHPG stimulation and induction of LTD in hippocampal CA1 (Waung et al., 2008). Consistent with an increase in translation, we see a significant increase in *Arc* mRNA in TRAP IPs isolated from DHPG-stimulated slices versus unstimulated controls (^∗^p = 0.016; Figure S4). Next, we examined whether DHPG lead to a similar recruitment of mAChR mRNAs to the ribosome-bound TRAP IP. Interestingly, our results show a robust increase in the expression of *Chrm4,* but not *Chrm1* or *Chrm3*, in the TRAP IP fraction upon DHPG stimulation (^∗^p = 0.0047; Figure 4B). No changes were seen in the corresponding Input fractions. These results show that *Chrm4* is selectively translated downstream of mGlu_1/5_ activation in CA1 pyramidal neurons.

To observe whether DHPG could increase *Chrm4* translation in the *Fmr1*^*−/y*^, we repeated our experiments on hippocampal slices from *Fmr1*^*−/y*^ and WT littermates. Our results reveal that while DHPG increases *Chrm4* in WT (^∗^p = 0.0047), it fails to do so in the *Fmr1*^*−y*^ slices where it is already elevated (WT versus KO veh ^∗^p = 0.048; KO veh versus DHPG p > 0.999; Figure 4C). Consistent with our previous experiments, *Chrm1* mRNA is enriched in the *Fmr1*^*−/y*^ TRAP pulldown (^∗^p = 0.046) but does not change with DHPG stimulation in either WT or *Fmr1*^*−/y*^ slices. *Chrm3* mRNA is neither increased in the *Fmr1*^*−/y*^ CA1-TRAP nor changed with DHPG (Figure 4C). To determine whether the increased translation of *Chrm4* upon DHPG stimulation leads to an increased expression of the M_4_ receptor, we measured the expression of M_4_ in hippocampal slices after the 5 min of DHPG stimulation and at 30 min and 60 min post-stimulation. This analysis reveals that mGlu_1/5_ activation in WT slices results in a remarkable increase in M_4_ expression, which is observed as early as 5 min post-stimulation (^∗^p = 0.0001), and remains elevated at 30 min (^∗^p = 0.047) and 60 min (^∗^p = 0.0051) post-stimulation (Figure 4D; Figure S5). In contrast, the increased expression of M_4_ observed in *Fmr1*^*−/y*^ slices (^∗^p = 0.022) remains unchanged with DHPG stimulation (Figure 4D; Figure S5). Thus, like global protein synthesis, the production of M_4_ downstream of mGlu_1/5_ activation is mimicked and occluded in *Fmr1*^*−/y*^ hippocampus.

Previous work shows that the exaggerated protein synthesis in the *Fmr1*^*−/y*^ hippocampus is sensitive to acute antagonism of mGlu_5_ (Osterweil et al., 2010). To investigate whether antagonism of mGlu_5_ could reduce the excess translation of M_4_, we incubated hippocampal slices in the selective mGlu_5_ antagonist 3-((2-Methyl-1,3-thiazol-4-yl)ethynyl)pyridine hydrochloride (MTEP) (Figure 4E) (Cosford et al., 2003). Our results show that application of 10 μM MTEP is sufficient to significantly reduce the level of *Chrm4* in *Fmr1*^*−/y*^ CA1-TRAP to WT levels (ANOVA genotype × treatment ^∗^p = 0.0119; KO veh versus KO MTEP ^∗^p = 0.0289; n = 8) (Figure 4F). To examine whether the reduction in *Chrm4* translation was reflected in a reduced expression in M_4_ protein, we performed quantitative immunoblotting of MTEP-treated slices. Consistent with our TRAP results, these experiments show that MTEP reduces M_4_ expression in the *Fmr1*^*−/y*^ hippocampus to WT levels (ANOVA genotype ^∗^p = 0.034; KO veh versus KO MTEP ^∗^p = 0.0145; n = 6) (Figure 4G; Figure S5). These findings confirm that the excess synthesis of M_4_ in the *Fmr1*^*−/y*^ hippocampus is downstream of mGlu_5_ activation.

### Positive Modulation of M_4_ Corrects Excessive Protein Synthesis in the *Fmr1*^*−/y*^ Hippocampus

**Figure 5:**
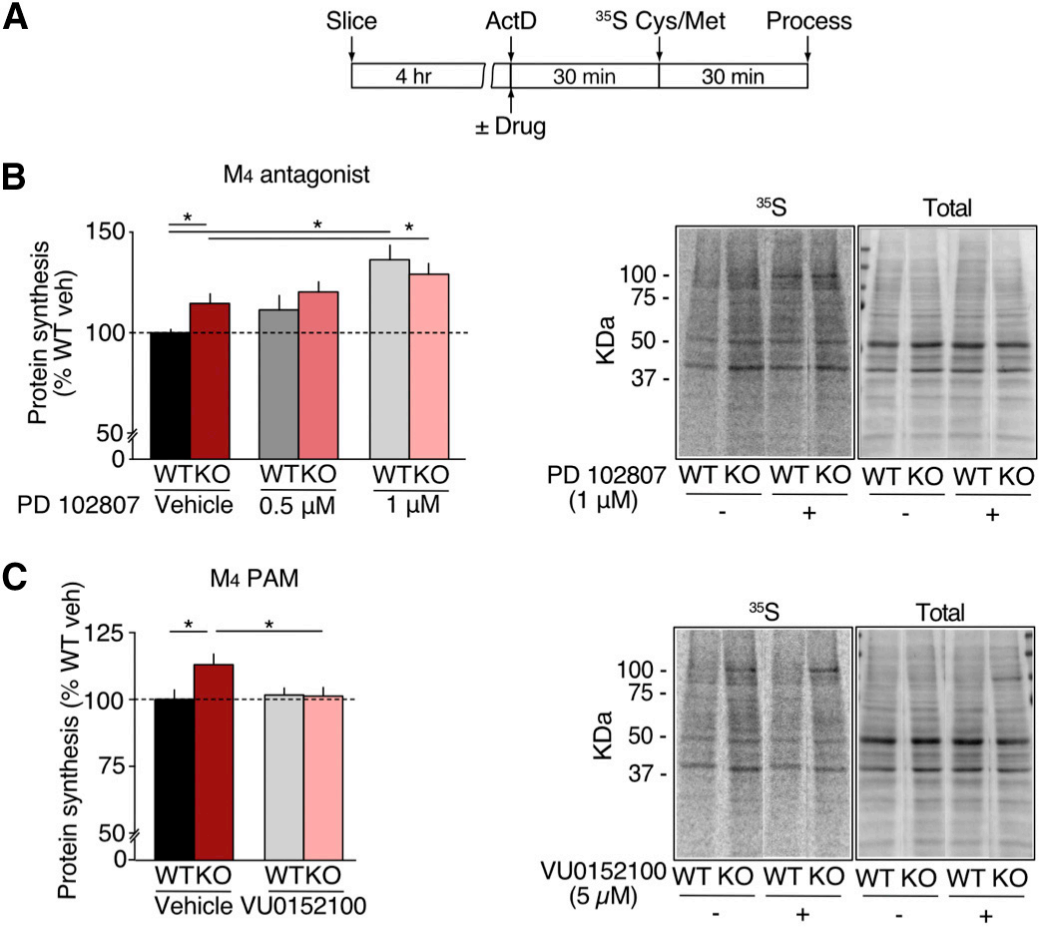
Enhancement of M_4_ Normalizes Excessive Protein Synthesis in the *Fmr1*^*−/y*^ Hippocampus (A) Time course for metabolic labeling experiments. (B) Treatment with the M_4_ antagonist PD 102807 (0.5 μM or 1 μM) significantly increases protein synthesis in both WT and *Fmr1*^*−/y*^ slices (WT vehicle = 100% ± 1.66%, WT PD 0.5 μM = 111.35% ± 7.16%, WT PD 1 μM = 136% ± 7.17%, KO vehicle = 114.59% ± 4.77%, KO PD 0.5 μM = 120.29% ± 5.02%, KO PD 1 μM = 129.05% ± 5.40%, ANOVA treatment ^∗^p < 0.0001, WT veh versus KO veh ^∗^p = 0.0379, WT veh versus WT PD 1 μM ^∗^p = 0.0009, KO veh versus KO PD 1 μM ^∗^p = 0.0065, n = 8). Example autoradiograph of slice homogenates shows upregulation of ^35^S-labeled proteins with M_4_ antagonist. Total protein stain of the same blot is shown for comparison. (C) Enhancement of M_4_ with VU0152100 (5 μM) results in selective reduction of protein synthesis in the *Fmr1*^*−/y*^ hippocampus, but no change in WT (WT veh = 100% ± 3.12%, KO veh = 114.2% ± 3.47%, WT VU = 101.7% ± 2.33%, KO VU = 101.3% ± 3.05%, ANOVA genotype × treatment ^∗^p = 0.0456, WT veh versus VU p = 0.6580, KO veh versus VU ^∗^p = 0.013, n = 16). Example autoradiograph shows a reduction of ^35^S-labeled proteins in *Fmr1*^*−/y*^ slices upon incubation with M_4_ PAM. Total protein stain of the same blot is shown for comparison. n = number of littermate pairs. Error bars indicate SEM.

The excessive translation of M_4_ in the *Fmr1*^*−/y*^ and correction by mGlu_5_ antagonism suggested that this receptor could be contributing to pathological changes in the *Fmr1*^*−/y*^ brain. To investigate this idea, we tested the effect of M_4_ antagonism on the excessive protein synthesis phenotype in the *Fmr1*^*−/y*^ hippocampus using an established metabolic labeling assay (see STAR Methods) (Osterweil et al., 2010). Briefly, hippocampal slices were prepared from *Fmr1*^*−/y*^ and WT littermates and new protein synthesis was measured in the presence of vehicle or the selective M_4_ antagonist PD 102807 by incorporation of ^35^S-methionine/cysteine (Figure 5A) (Olianas and Onali, 1999). Surprisingly, our results showed that application of PD 102807, at doses previously shown to be selective for M_4_, caused a significant increase in protein synthesis in both WT and *Fmr1*^*−/y*^ slices (ANOVA treatment ^∗^p < 0.0001; WT veh versus KO veh ^∗^p = 0.0379; WT veh versus WT PD 1 μM ^∗^p = 0.0009, KO veh versus KO PD 1 μM ^∗^p = 0.0065; n = 8) (Figure 5B) (Stoll et al., 2009). This indicates that M_4_ antagonism worsens the excessive protein synthesis phenotype in the *Fmr1*^*−/y*^ hippocampus. In contrast, application of the M_1_-specific antagonist pirenzepine (75 nM) significantly reduces protein synthesis; however, this does not correct the difference between WT and *Fmr1*^*−/y*^ hippocampi (ANOVA genotype ^∗^p = 0.0183, treatment ^∗^p = 0.0119; WT veh versus Pz ^∗^p = 0.021; KO versus Pz ^∗^p = 0.048; Figure S6). Thus, inhibition of neither M_4_ nor M_1_ normalizes the excessive protein synthesis phenotype in the *Fmr1*^*−/y*^ hippocampus.

The robust overexpression of M_4_ in the *Fmr1*^*−/y*^ and its selective translation downstream of mGlu_5_ strongly suggested an involvement in FX pathology. Given that M_4_ inhibition did not resolve the protein synthesis phenotype, we wondered whether the increased synthesis of M_4_ in *Fmr1*^*−/y*^ neurons represented a compensatory change rather than a direct cause of altered function. To test this hypothesis, we obtained a highly selective M_4_ PAM, VU0152100, which enhances the effects of cholinergic agonists on M_4_ without impacting other mAChRs (Brady et al., 2008). To test whether M_4_ enhancement could correct excessive protein synthesis in the *Fmr1*^*−/y*^, we incubated slices in 5 μM VU0152100, a concentration shown to specifically enhance M_4_ function in acute brain slices (Pancani et al., 2014). Remarkably, our results show that VU0152100 significantly reduces the level of protein synthesis in the *Fmr1*^*−/y*^ while having no effect on the WT hippocampus (ANOVA genotype × treatment ^∗^p = 0.0456, WT veh versus VU p = 0.658; KO veh versus VU ^∗^p = 0.0135; Figure 5C). The surprising conclusion is that enhancement of M_4_, a protein over-translated and overexpressed in the *Fmr1*^*−/y*^ hippocampus, corrects the exaggerated protein synthesis phenotype.

In addition to exaggerated protein synthesis, a prominent cellular change observed in the *Fmr1*^*−/y*^ is a reduced production of cAMP upon stimulation of adenylate cyclase (AC) (Berry-Kravis et al., 1995, Berry-Kravis and Huttenlocher, 1992, Kelley et al., 2007). The relevance of this phenotype to the pathology of FX is confirmed by experiments that show that increasing cAMP production corrects several behavioral measures of learning in multiple animal models (Choi et al., 2015, Choi et al., 2016). As the M_4_ receptor is coupled to the AC/cAMP pathway, we wondered whether the normalization of protein synthesis by M_4_ PAM was due to a change in cAMP signaling. To test this, we stimulated *Fmr1*^*−/y*^ and WT slices with the potent AC activator forskolin (FSK; 50 μM) in the presence of vehicle or VU0152100 (see STAR Methods; Figure S7). Consistent with previous studies, we find a significant deficit in FSK-stimulated cAMP production in the *Fmr1*^*−/y*^ (ANOVA treatment ^∗^p < 0.0001, genotype × treatment ^∗^p = 0.0264, WT FSK versus KO FSK ^∗^p = 0.0214, n = 9). However, application of VU0152100 had no effect on the stimulation of cAMP in either WT (p < 0.306) or *Fmr1*^*−/y*^ (p < 0.4625, n = 9; Figure S7) slices. These results suggest that although it may be involved in other phenotypes, the stimulation of cAMP downstream of M_4_ activation is not responsible for the correction of protein synthesis by M_4_ PAM.

### M_4_ PAM Corrects the Exaggerated mGluR-LTD Phenotype in *Fmr1*^*−/y*^ CA1

**Figure 6:**
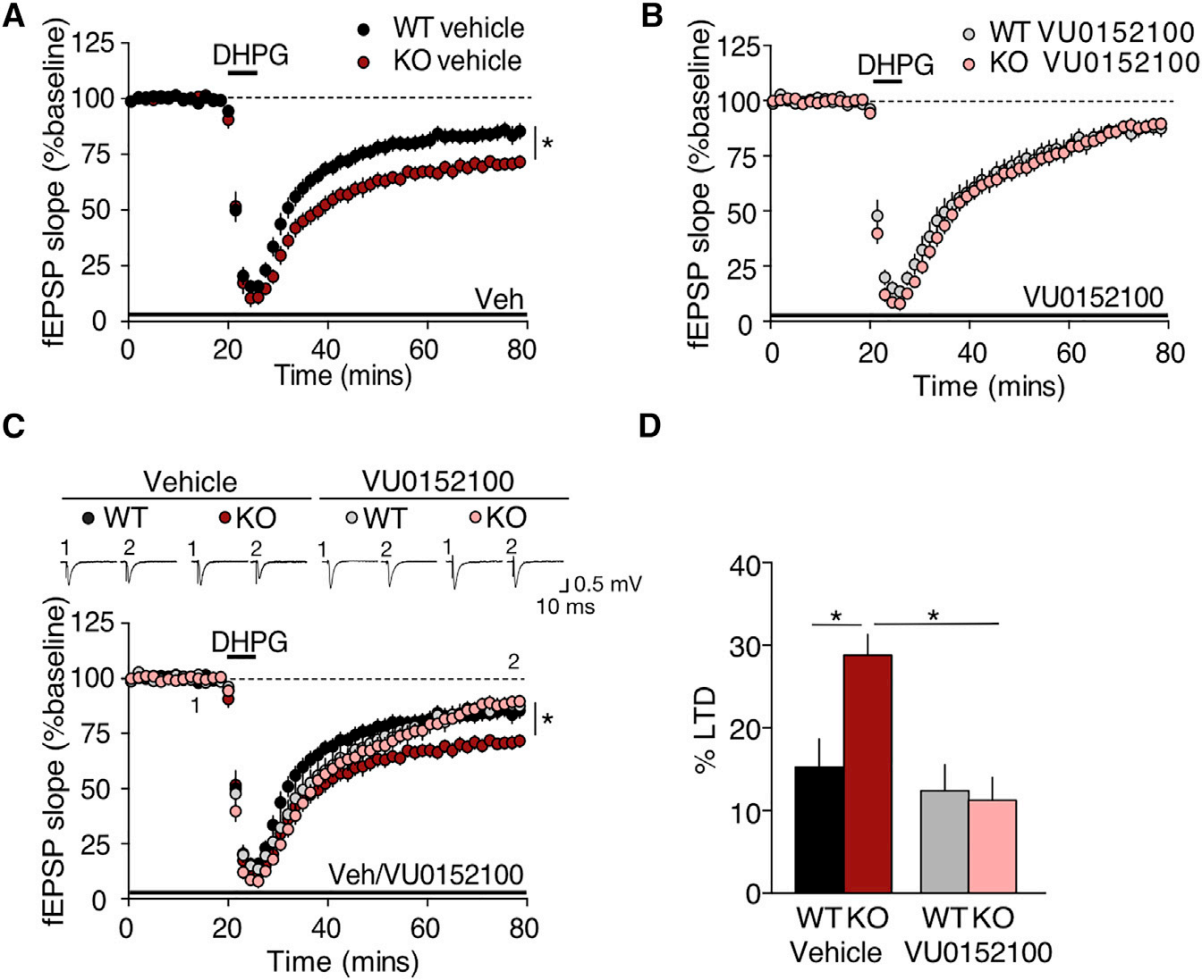
M_4_ PAM Corrects Exaggerated mGluR-LTD in the *Fmr1*^*−/y*^ Mouse (A) Measurement of mGluR-LTD in hippocampal CA1 shows a significant elevation in vehicle-treated *Fmr1*^*−/y*^ versus WT (WT = 84.7% ± 3.4%, n = 16, KO = 71.2% ± 2.47%, n = 15, *^∗^*p = 0.0028). (B) Exaggerated LTD in the *Fmr1*^*−/y*^ is significantly normalized with 5 μM VU0152100 (KO PAM = 88.7% ± 2.76%, n = 13, ^∗^p = 0.0003). VU0152100 treatment has no effect on WT LTD (WT PAM 87.6% ± 3.13%, n = 11, p > 0.999). (C) Comparison of all four groups (re-plotted from A and B). (D) Quantification of the last 10 min of recording shows a significant rescue of the LTD phenotype in the *Fmr1*^*−/y*^ with VU0152100 (ANOVA genotype × treatment ^∗^p = 0.0191). n = number of animals. Error bars indicate SEM.

Based on the beneficial effect seen in our biochemical assays, we wondered whether M_4_ enhancement could also correct the exaggerated mGluR-LTD in *Fmr1*^*−/y*^ CA1. To test this, we performed extracellular recordings in the CA1 region of hippocampal slices ±5 μM VU0152100. LTD was stimulated using 50 μM *S*-DHPG, and recordings were performed on hippocampal slices prepared from WT and *Fmr1*^*−/y*^ littermates, consistent with previous work (Barnes et al., 2015). In keeping with previous findings, our results show a significant enhancement of mGluR-LTD in vehicle-treated *Fmr1*^*−/y*^ slices (*^∗^*p = 0.0028; Figure 6A) (Huber et al., 2002). However, the application of VU0152100 resulted in a striking reduction of the exaggerated LTD phenotype in *Fmr1*^*−/y*^ slices (^∗^p = 0.0003) without affecting LTD magnitude in WT slices. A comparison of LTD in WT and KO VU0152100-treated slices reveals no significant difference (Figure 6B). These results show that positive modulation of M_4_ with VU0152100 resolves exaggerated mGluR-LTD in the *Fmr1*^*−/y*^ hippocampus (ANOVA genotype × treatment ^∗^p = 0.0191; Figures 6C and 6D), further supporting the idea that *Chrm4* over-translation is a protective mechanism in the *Fmr1*^*−/y*^ hippocampus.

### M_4_ PAM Corrects AGS in the *Fmr1*^*−/y*^ Mouse

The positive effects of VU0152100 on the biochemical and electrophysiological phenotypes in *Fmr1*^*−/y*^ motivated us to test this treatment *in vivo*. One of the most robust behavioral phenotypes observed in the *Fmr1*^*−/y*^ mouse model is an increased susceptibility for AGS (Yan et al., 2005). Treatments that correct this core phenotype have been found to be effective in ameliorating many other pathological changes in FX (Michalon et al., 2012, Osterweil et al., 2013, Yan et al., 2005). To test whether M_4_ PAM could also correct the AGS phenotype, we injected *Fmr1*^*−/y*^ and WT littermates with vehicle (10% DMSO + 10% Tween-80 in PBS) or 56 mg/kg VU0152100, a dose previously shown to be effective in reducing aberrant behaviors in mouse and rat models of psychosis while having no effect on M_4_ KO mice (Byun et al., 2014).

**Figure 7:**
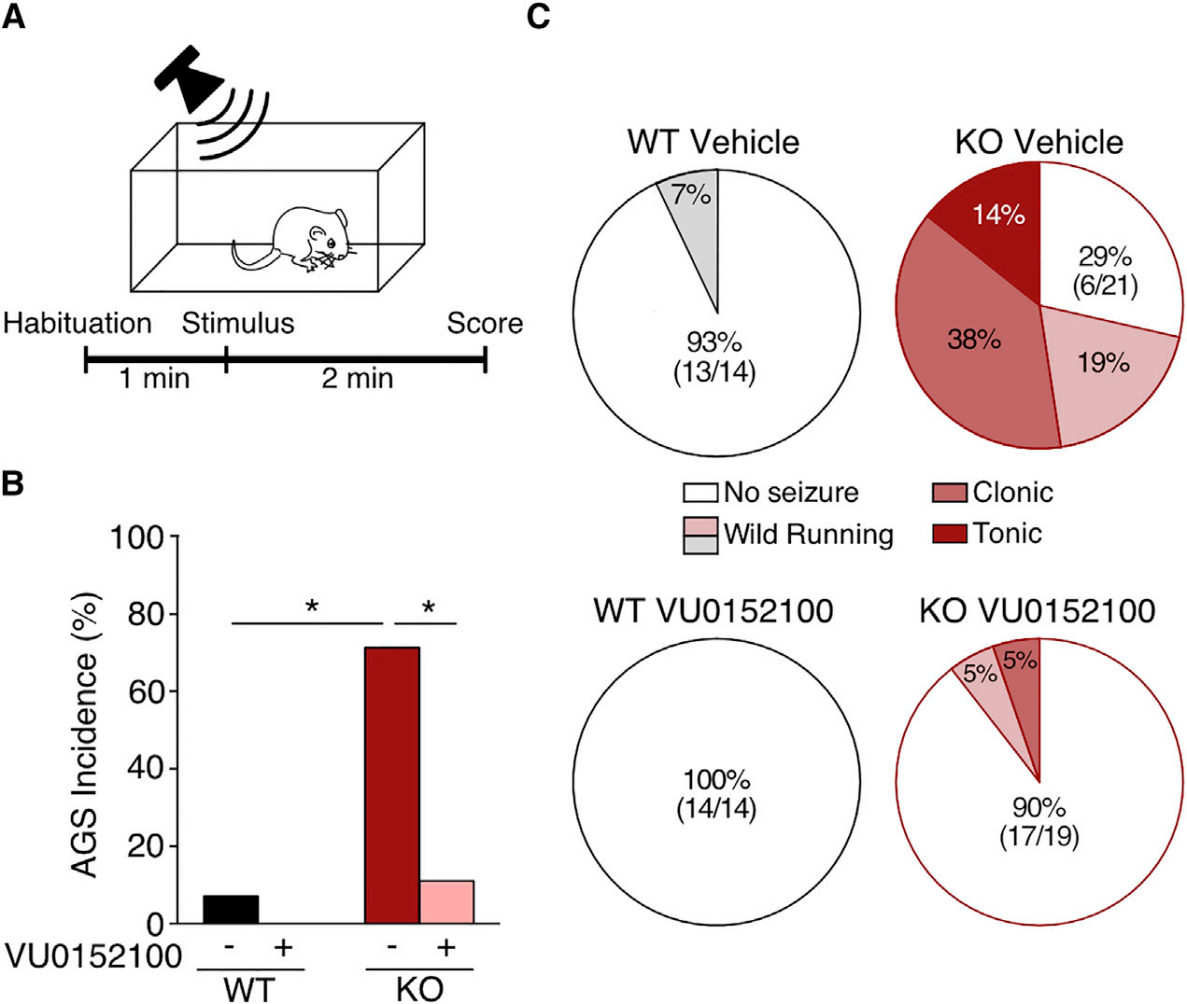
M_4_ PAM Corrects the Exaggerated AGS Phenotype in the *Fmr1*^*−/y*^ Mouse (A) Time course for AGS experiments. (B) Injection of VU0152100 significantly reduces the incidence of AGS in *Fmr1*^*−/y*^ mice versus vehicle (Fisher’s exact test ^∗^p < 0.0001; KO veh 15/21, KO VU 2/19, WT veh 1/14, WT PAM 0/14). (C) VU0152100 reduces severity of AGS in the *Fmr1*^*−/y*^ (KO veh wild running 4/21, clonic 11/21, tonic 3/21; KO VU wild running 1/19, clonic 1/19).

To test for AGS, we habituated animals to the testing arena and then exposed them to a loud (>120 dB) alarm for 2 min (see STAR Methods; Figure 7A). If seizures occurred, they were scored for increasing stages of severity: wild running (pronounced, undirected running, and thrashing), clonic seizure (violent spasms accompanied by loss of balance), or tonic seizure (postural rigidity in limbs). In accordance with previous work, we find that vehicle-treated *Fmr1*^*−/y*^ mice exhibit a 71% incidence of AGS (15/21 animals versus 1/14 animals for WT, ^∗^p < 0.0001; Figure 7B). Remarkably, injection of VU0152100 reduces this incidence to 10% (2/19 animals, ^∗^p < 0.0001). This treatment also reduces the severity of AGS in *Fmr1*^*−/y*^ mice, lowering the incidence of clonic seizures from 38% (11/21) to 5% (1/19) and eliminating tonic seizures (Figure 7C). Thus, injection of VU0152100 significantly reduces both the incidence and severity of AGS in the *Fmr1*^*−/y*^. Together, our results suggest that positive enhancement of M_4_ is corrective for multiple *Fmr1*^*−/y*^ phenotypes.

## Discussion

**Figure 8:**
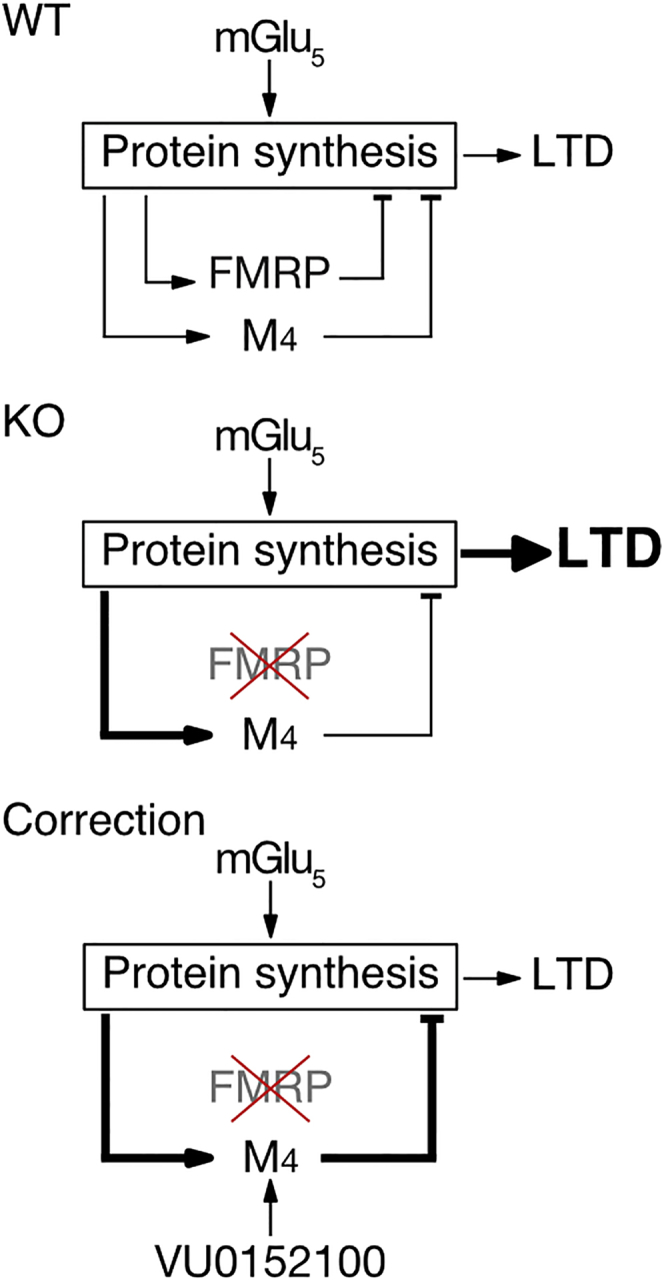
Potential Model for Correction of FX by M_4_ PAM Our results suggest a model whereby M_4_ is synthesized downstream of mGlu_5_ in order to negatively regulate protein synthesis and LTD, similar to FMRP. In FX, the absence of FMRP leads to the excessive synthesis of M_4_; however, this is unable to completely compensate for FMRP loss. By enhancing M_4_ with VU0152100, protein synthesis, LTD, and other pathological changes are normalized.

In this study, we sought to tie pathological changes in FX to the altered translation of specific mRNAs. We chose to do this in a cell-type-specific way so that we could isolate molecular changes that could be interrogated at the physiological level. Our results reveal an increase in the translation of *Chrm4* and *Chrm1* mRNA and overexpression of M_4_ and M_1_ receptors in *Fmr1*^*−/y*^ CA1 neurons. An mGluR-LTD induction protocol stimulates the translation of *Chrm4* and expression of M_4_ in WT CA1 neurons; however, this is saturated in the *Fmr1*^*−/y*^ hippocampus. Application of the mGlu_5_ antagonist MTEP normalizes M_4_ translation in the *Fmr1*^*−/y*^ hippocampus. Surprisingly, although it is excessively translated in the *Fmr1*^*−/y*^, antagonism of M_4_ worsens the protein synthesis phenotype. In contrast, positive modulation of M_4_ using the selective PAM VU0152100 corrects core phenotypes in the *Fmr1*^*−/y*^, including excessive protein synthesis, exaggerated mGluR-LTD, and increased susceptibility to AGS. The startling conclusion is that enhancing M_4_, a protein over-translated and overexpressed in the *Fmr1*^*−/y*^, is a potential new strategy for correcting FX neuropathology (Figure 8).

Although the TRAP method was developed to identify differences in the expression of mRNAs in select neuronal populations, with no distinction between changes driven by translation or transcription (Doyle et al., 2008, Heiman et al., 2008), we used this approach to investigate the increased translation that is a well-known pathophysiology in the *Fmr1*^*−/y*^ mouse. While we cannot rule out the possibility that some of the changes we observe by RNA-seq are due to total mRNA expression differences specific to CA1 neurons, our measurement of total *Camk2a*, *Chrm1*, or *Chrm4* mRNAs in FACS-isolated CA1 neurons reveal no differences between *Fmr1*^*−/y*^ and WT despite a robust increase in the *Fmr1*^*−/y*^ TRAP IP (Figures 1E, 3A, and 3B).

Upon examination of the differentially expressed transcripts, we discovered that FMRP targets were not enriched in the *Fmr1*^*−/y*^ CA1-TRAP fraction (Figure 2D). Further investigation found that the cumulative distribution of FMRP targets was in fact reduced in both the starting Input fraction, comprised of total hippocampal mRNAs, and the CA1-TRAP fraction. While this is seemingly inconsistent with the mechanism of FMRP as a repressor of translation, it may be that the loss of FMRP early in development results in a homeostatic downregulation of FMRP target mRNAs. It is also possible that loss of FMRP disrupts RNA transport and/or stability (Feng et al., 1997, Tamanini et al., 1999, Zalfa et al., 2007). Future experiments investigating how the loss of FMRP results in the eventual dysregulation of its target mRNAs in the *Fmr1*^*−/y*^ brain should be particularly interesting.

A comparative analysis of WT versus *Fmr1*^*−/y*^ CA1-TRAP revealed 121 differentially expressed transcripts at FDR < 0.1 (Tables S1 and S2). This significance cutoff has been used in previous RNA-seq studies (Cho et al., 2015, Tao et al., 2016), and it allowed us to include genes that would otherwise have been excluded as false negatives. Although several genes on this list may be revealed to be relevant to the synaptic dysfunction in FX, our unbiased analyses of enriched cellular pathways and drug interaction targets pointed to *Chrm4* as the most obvious candidate for further investigation (Figures 2G and 2H). The elevated translation of *Chrm4* and *Chrm1* in the *Fmr1*^*−/y*^ hippocampus is particularly interesting in light of previously published studies. In particular, an enhancement of LTD downstream of M_1_ has been observed in the *Fmr1*^*−/y*^ hippocampus (Volk et al., 2007), and some of the behavioral effects seen in the *Fmr1*^*−/y*^ are ameliorated by M_1_ inhibitors (Veeraragavan et al., 2011). Our results suggest these effects may be due, at least in part, to an overexpression of M_1_. In contrast, genetic reduction of M_4_ does not appear to correct cognitive deficits (Veeraragavan et al., 2012), which is consistent with our results showing that M_4_ antagonism does not correct protein synthesis in the *Fmr1*^*−/y*^ hippocampus (Figure 5B). Whether the enhancement of M_4_ can improve cognitive phenotypes in the *Fmr1*^*−/y*^ is an open question; however, the correction of AGS by VU0152100 shows that positive modulation of M_4_ has a beneficial impact on brain circuits other than hippocampal CA1 the *Fmr1*^*−/y*^ mouse. It will be interesting to investigate the more widespread effects of M_4_ PAM on other neuronal populations in the *Fmr1*^*−/y*^ brain.

Our model suggests that the positive modulation of M_4_ corrects LTD in the *Fmr1*^*−/y*^ hippocampus by reducing excess protein synthesis downstream of mGlu_5_ (Figure 8). Several other strategies that acutely reduce protein synthesis have also been shown to correct LTD in the *Fmr1*^*−/y*^, including mGlu_5_ antagonist, lovastatin, and lithium (Choi et al., 2011, Michalon et al., 2012, Osterweil et al., 2013). However, these results are seemingly inconsistent with studies showing that a complete inhibition of protein synthesis does not block LTD in the *Fmr1*^*−/y*^ hippocampus (Nosyreva and Huber, 2006). Although the explanation for this is currently unknown, the idea that partial inhibition of protein synthesis differs from a complete block of translation is not entirely unprecedented. Indeed, previous experiments in isolated synaptic fractions shows that partial block of translation with low-dose cycloheximide paradoxically increases the translation of specific mRNAs while inhibiting global translation (Scheetz et al., 2000). It may be that partial reduction of protein synthesis in the *Fmr1*^*−/y*^ restores the translation of the mRNAs needed to support normal levels of LTD. Alternatively, it is possible that complete inhibition of protein synthesis in the *Fmr1*^*−/y*^ triggers changes in other cellular processes, such as protein breakdown or mRNA decay, which facilitates LTD (Harper and Bennett, 2016). To distinguish between these possibilities, further mechanistic studies are needed to fully understand the relationship between mRNA translation and LTD at *Fmr1*^*−/y*^ synapses.

Reduction of excessive protein synthesis by inhibiting mGlu_5_ or the downstream ERK pathway have been shown to be successful strategies for correcting pathological changes in the *Fmr1*^*−/y*^ mouse (Stoppel et al., 2017). However, recent attempts to transition mGlu_5_ antagonists into a clinical setting have not been successful. Thus, it has become increasingly important to identify alternative treatment strategies that more specifically target the dysregulated translation downstream of mGlu_5_. Our results show that M_4_ is synthesized downstream of mGlu_5_, acting as a protective mechanism that can be enhanced using the M_4_ PAM VU0152100 (Figure 8). M_4_ PAMs have been proposed as a treatment for multiple neuropsychiatric disorders, including schizophrenia and Alzheimer’s disease (Jones et al., 2012). Studies in rodents have shown that the administration of M_4_ PAMs result in pro-cognitive effects,without causing negative side effects associated with less specific cholinergic modulators (Brady et al., 2008, Bubser et al., 2014). Thus, M_4_ PAMs may represent a novel treatment option.

Perhaps more importantly, our study shows that not all excessively translating mRNAs in FX are contributing to pathological changes. Many studies have focused on reducing the expression of FMRP target mRNAs in the *Fmr1*^*−/y*^, following the assumption that this will correct phenotypes. However, our results show that the reverse approach is successful in the case of M_4_. This raises the possibility that other excessively translating mRNAs may similarly be protective adaptations. This does not argue against the idea that excessive protein synthesis is pathological, but it does suggest that the specific mRNAs translating in excess are important to evaluate. Indeed, enhancing the function of certain over-synthesized proteins may be an overlooked approach to correcting FX.

## STAR★Methods

### Key Resources Table

**Table:** 

REAGENT or RESOURCE	SOURCE	IDENTIFIER
**Antibodies**		

Muscarinic acetylcholine receptor M3 antibody	GeneTex	GTX111637; RRID: AB_11167520
Anti-Muscarinic Acetylcholine Receptor (M1) antibody produced in rabbit, affinity isolated antibody, lyophilized powder	Sigma	M9808-.2ML; RRID: AB_260731
Anti-Muscarinic Acetylcholine Receptor M4 antibody (18C7.2)	Abcam	ab77956; RRID: AB_1566454
HtzGFP-19F7	Memorial Sloan Kettering Center	HtzGFP-19F7
HtzGFP-19C8	Memorial Sloan Kettering Center	HtzGFP-19C8
Anti-rabbit IgG, HRP-linked Antibody	Cell Signaling Technology	7074; RRID: AB_2099233
Anti-mouse IgG, HRP-linked Antibody	Cell Signaling Technology	7076; RRID: AB_330924
Donkey anti-Rabbit IgG Secondary Antibody, Alexa 594	Thermo Fisher Scientific	R37119; RRID: AB_2556547
Donkey anti-Mouse IgG Secondary Antibody, Alexa 594	Thermo Fisher Scientific	R37115; RRID: AB_2556543

**Chemicals, Peptides, and Recombinant Proteins**		

S-DHPG	Sigma	D3689
MTEP hydrochloride	Tocris	2921
Actinomycin D	Tocris	1229
PD102807	Tocris	1671
VU0152100	Sigma	V5015
Pirenzepine dihydrochloride	Tocris	1071
EasyTag EXPRESS35S Protein Labeling Mix, [35S]−, 14mCi (518MBq), Stabilized Aqueous Solution	PerkinElmer	NEG772014MC
Forskolin	Sigma	F6886
IBMX	Sigma	I5879

**Critical Commercial Assays**		

Absolutely RNA Nanoprep Kit	Agilent	400753
Quant-iT RiboGreen RNA Assay Kit	Thermo Fisher Scientific	R11490
RNA 6000 Pico Kit	Agilent	5067-1513
Superscript VILO cDNA Synthesis Kit	Thermo Fisher Scientific	11755050
Quantitect SYBRgreen qPCR master mix	QIAGEN	204143
RNaseq Ovation V2 kit	NuGEN	7102
Pierce Reversible Protein Stain Kit for Nitrocellulose Membranes	Thermo Fisher Scientific	24580
Clarity Western ECL Substrate	Bio-Rad	1705061
DC Protein Assay Kit II	Bio-Rad	5000112
cAMP—Gs Dynamic kit—1,000 tests	Cisbio	62AM4PEB

**Deposited Data**		

RNA sequencing data	This paper	GEO: GSE101823

**Experimental Models: Organisms/Strains**		

*Fmr1*^*−/y*^	The Jackson Laboratory	004624; RRID: IMSR_JAX:004624
CA1-TRAP	The Jackson Laboratory	GM391-TRAP

**Oligonucleotides**		

GACAACTTTGGCATTGTGGA	IDT	Gapdh Forward
CATCATACTTGGCAGGTTTCTC	IDT	Gapdh Reverse
GGAATCTTCTGAGAGCACCA	IDT	Camk2a Forward
CACATCTTCGTGTAGGACTC	IDT	Camk2a Reverse
CCATCAACATGCTCCCGTTC	IDT	Wfs1 Forward
GGGTAGGCCTCGCCATACA	IDT	Wfs1 Reverse
CACAGGTCACCCTCGATTTTT	IDT	Gad1 Forward
ACCATCCAACGATCTCTCTCATC	IDT	Gad1 Reverse
TCCTGGAACAGCAAAACAAG	IDT	Gfap Forward
CAGCCTCAGGTTGGTTTCAT	IDT	Gfap Reverse
TCTCTGAATGCTGGAAGTAAAGA	IDT	Chrm1 Forward
GAGACCCTAGATTCAGTCCCA	IDT	Chrm1 Reverse
AGGGCTGACTACTTAATCTTGGATA	IDT	Chrm3 Forward
TGCAAGGTCATTGTGACTCTC	IDT	Chrm3 Reverse
CAGCGGAGCAAGACAGAAG	IDT	Chrm4 Forward
GCACAGACTGATTGGCTGAG	IDT	Chrm4 Reverse

**Software and Algorithms**		

GraphPad Prism v.6	GraphPad Software	N/A
Microsoft Excel	Microsoft	N/A
R	R-project	N/A
STAR 2.4.0i	Dobin et al., 2013	N/A
featureCounts 1.4.6-p2	Liao et al., 2014	N/A
FlowjJ v.10	FlowJo	N/A

### Contact for Reagent and Resource Sharing

Further information and requests for resources and reagents should be directed to and will be fulfilled by the Lead Contact, Emily Osterweil (emily.osterweil@ed.ac.uk). The CA1-TRAP mouse (GM391-TRAP) was obtained from a repository at Jackson Labs, and antibodies for TRAP (HtzGFP-19F7 and HtzGFP-19C8) were obtained from Sloan Memorial Kettering Centre, after establishing MTAs with the laboratory of Prof. Nathaniel Heintz at The Rockefeller University.

### Experimental Model and Subject Details

#### Mice

*Fmr1*^*-/y*^ and CA1-TRAP mice (created by http://gensat.org/ and obtained from Jackson Labs with permission from Nathanial Heintz) were bred on the JAX C57BL/6J background. All experiments were carried out using male littermate mice aged P25-32, and studied with the experimenter blind to genotype. *Fmr1*^*-/y*^ and WT littermates were bred using *Fmr1*^*+/−*^ females and JAX C57BL/6J males. *Fmr1*^*-/y*^-TRAP and WT-TRAP littermates were bred using *Fmr1*^*+/−*^ females and CA1-TRAP homozygous males.

All mice were naive to drug and behavioral testing. Mice were group housed (6 maximum) in conventional non-environmentally enriched cages with unrestricted food and water access and a 12h light-dark cycle. Room temperature was maintained at 21 ± 2°C. Animal husbandry was carried out by University of Edinburgh technical staff. All procedures were in performed in accordance with ARRIVE guidelines and the regulations set by the University of Edinburgh and the UK Animals Act 1986.

### Method Details

#### Confocal Imaging

CA1-TRAP mice were perfused with 4% PFA and 50 μm coronal vibratome sections mounted with Vectashield (Vector labs) and imaged by confocal microscope (Nikon A1R FILM) in collaboration with the IMPACT facility at the University of Edinburgh.

#### TRAP

Briefly, CA1-TRAP WT and *Fmr1*^*-/y*^ male littermates (P25-32) were decapitated and hippocampi rapidly dissected in ice cold PBS. Hippocampi were homogenized in ice-cold lysis buffer (20 mM HEPES, 5 mM MgCl_2_, 150 mM KCl, 0.5 mM DTT, 100 μg/ml cyclohexamide, RNase inhibitors and protease inhibitors) using dounce homogenizers, and samples centrifuged at 1,000 x g for 10 min to remove large debris. Supernatants were then extracted with 1% NP-40 and 1% DHPC on ice, and centrifuged at 20,000 x g for 20 min. A 50 μL sample of supernatant was removed for use as Input, and the rest incubated with streptavidin/protein L-coated Dynabeads (Life Technologies) bound to anti-GFP antibodies (HtzGFP-19F7 and HtzGFP-19C8, Memorial Sloan Kettering Centre) overnight at 4°C with gentle mixing. Anti-GFP beads were washed with high salt buffer (20 mM HEPES, 5 mM MgCl_2_, 350 mM KCl, 1% NP-40, 0.5 mM DTT and 100 μg/ml cyclohexamide) and RNA was eluted from all samples using Absolutely RNA Nanoprep kit (Agilent) according to the manufacturer’s instructions. RNA yield was quantified using RiboGreen (Life Technologies) and RNA quality was determined by Bioanalyzer analysis.

#### RT-qPCR

RNA for each sample was converted into cDNA using Superscript VILO cDNA Synthesis Kit (Life Technologies) and RT-qPCR was performed using Quantitect SYBRgreen qPCR master mix (QIAGEN) according to the manufacturer’s instructions. Samples were prepared in triplicate in 96-well reaction plates and run on a StepOne Plus (Life Technologies). For TRAP analysis, each sample was normalized to *Gapdh*, and then each IP was normalized to the corresponding Input sample. For FACS analyses, all samples were first normalized to *Gapdh,* and then each GFP-positive or GFP-negative sample was normalized to the corresponding sample from all cells. Primers used for RT-qPCR are as follows: *Gapdh* (F- GACAACTTTGGCATTGTGGA, R- CATCATACTTGGCAGGTTTCTC); *Camk2a* (F- GGAATCTTCTGAGAGCACCA, R- CACATCTTCGTGTAGGACTC); *Wfs1* (F- CCATCAACATGCTCCCGTTC, R- GGGTAGGCCTCGCCATACA); *Gad1* (F- CACAGGTCACCCTCGATTTTT, R- ACCATCCAACGATCTCTCTCATC); *Gfap* (F- TCCTGGAACAGCAAAACAAG, R- CAGCCTCAGGTTGGTTTCAT); *Chrm1* (F- TCTCTGAATGCTGGAAGTAAAGA, R- GAGACCCTAGATTCAGTCCCA); *Chrm3* (F- AGGGCTGACTACTTAATCTTGGATA, R- TGCAAGGTCATTGTGACTCTC); *Chrm4* (F- CAGCGGAGCAAGACAGAAG, R- GCACAGACTGATTGGCTGAG); *Chrm5* (F- TTAAGCTGCTGCTTCTCTGC, R- TTTCCAGAGGAGTTGCTAAGG); *Arc* (F- CAGGGGTGAGCTGAAGCCACAAA, R- CCATGTAGGCAGCTTCAGGAGAAGAGAG).

#### RNA-Seq

RNA with RIN > 7 was prepared for RNA-seq using the RNaseq Ovation V2 kit (Nugen), according to manufacturer’s instructions. Samples were sent to Oxford Genomics Centre for sequencing using Illumina HiSeq 2500 or HiSeq 4000. Sequencing reads (50 or 75 bp, paired end) were mapped to the *Mus musculus* primary assembly (Ensembl release v80) using STAR 2.4.0i (Dobin et al., 2013). Reads that were uniquely aligned to annotated genes were counted with featureCounts 1.4.6-p2 (Liao et al., 2014). Differential expression analyses were performed using DESeq2 1.12.4 with betaPrior = FALSE (Love et al., 2014). TPM (transcripts per million) values were determined using Salmon 0.7.2 at the transcript level and gene TPMs were calculated by adding the values of all transcripts for each gene (Patro et al., 2017). Cell type specificity analyses were performed with Enrichr using the Allen_brain_atlas_up library. A heatmap of differentially expressed genes was created using pheatmap 1.0.8. TPM values were normalized to the average TPM of the WT and the values were scaled before creating the heatmap. GO analyses were performed with Enrichr (http://amp.pharm.mssm.edu/Enrichr/) (Chen et al., 2013) using the GO_molecular_function 2015 library. Number of drugs interacting with Genes differentially regulated in the *Fmr1*^*-/y*^ CA1-TRAP were quantified using the Drug Gene Interaction database (http://dgidb.genome.wustl.edu/) (Griffith et al., 2013).

#### pClan Analysis

Genes differentially expressed in the *Fmr1*^*-/y*^ CA1-TRAP were categorized into pClans using the Pfam database (EMBL_EBI, http://pfam.xfam.org/). An updated list of FMRP targets (Jen Darnell, personal communication) was adjusted to include only genes identified in at least one CA1-TRAP IP (TPM > 0). Adjusted FMRP targets were also categorized into pClans using the Pfam database. pClans from these lists were compared with the pClans from the background list of all CA1-TRAP genes (TPM > 0 in any one sample) to identify enriched pClans. pClan enrichment (over background CA1-TRAP genes) was determined by Fisher’s Exact test (p < 0.01). Significantly enriched pClans were compared between *Fmr1*^*-/y*^ CA1-TRAP genes and FMRP targets.

#### FMRP Target Analysis

Genes with DESeq2 normalized counts similar to FMRP targets were selected for comparison from the differentially expressed Input and CA1-TRAP fractions (Input: between 10^2.5^ and 10^4.25^; CA1-TRAP: between 10^2.75^ and 10^4.75^). Cumulative distributions of log_2_ fold change were compared between FMRP targets and either all genes within the same abundance (Figure 2) or to 5 randomly selected gene sets of the same number (Figure S3). Significance determined by K-S test. In addition, the proportion of up- and downregulated genes was compared and significance determined by Fisher’s exact test (Figure S3).

#### Hippocampal Slice Preparation

Hippocampal slices were prepared from male littermate WT and *Fmr1*^*-/y*^ mice (P25-32), in an interleaved fashion, with the experimenter blind to genotype as described previously (Osterweil et al., 2010). Briefly, mice were anaesthetized with isofluorane and the hippocampus was rapidly dissected in ice-cold ACSF (124 mM NaCl, 3 mM KCl, 1.25 mM NaH_2_PO_4_, 26 mM NaHCO_3_, 10 mM dextrose, 1 mM MgCl_2_ and 2 mM CaCl_2_, saturated with 95% O_2_ and 5% CO_2_). Slices (500 μm thick) were prepared using a Stoelting Tissue Slicer and transferred into 32.5°C ACSF (saturated with 95% O_2_ and 5% CO_2_) within 5 min. Slices were incubated in ACSF for 4 hr to allow for recovery of protein synthesis. For DHPG stimulation, slices were transferred into ACSF containing 50 μM S-DHPG (Sigma) or vehicle (ddH_2_O) for 5 min, before being transferred to fresh ACSF to recover for an additional 25 or 55 min. For MTEP stimulations, slices were transferred to ACSF containing 10 μM MTEP (Tocris) or vehicle (ddH_2_O) for 1.5 hr. Slices were either processed for TRAP or immunoblotted.

#### Synaptoneurosome Preparation

Hippocampal slices were prepared as above, then homogenized in ice-cold homogenization buffer (10 mM HEPES, 2 mM EDTA, 2 mM EGTA, 150 mM NaCl) in 2 mL Dounce homogenizers. Homogenates were filtered through a 100 μm filter (Millipore), followed by a 5 μm filter (Millipore). Homogenates were centrifuged at 10000 x g for 10 min and the supernatant was discarded. The pellet was re-suspended in lysis buffer (50 mM HEPES, 5 mM EDTA, 150 mM NaCl, 1% Triton X-100, 0.5% sodium deoxycholate, 0.1% SDS) and protein concentrations were determined using BioRad DC (BioRad). Samples were boiled in Laemmli sample buffer for immunoblotting.

#### FACS

Hippocampal slices were prepared and recovered as above. CA1 was micro-dissected and incubated in ACSF with papain (20 U/ml; Sigma-Aldrich) for 45 min at 37°C with 5% CO_2_. Tissue was dissociated using a fire polished glass pipette and filtered using a 70 μm cell sieve. A sample of single cell dissociate was used to isolate RNA from all cell types prior to sorting. Cell sorting was performed on FACSAria II (BD bioscience) using DAPI as a live/dead marker. From each mouse, an average of 1500-GFP positive neurons or 10,000 GFP-negative cells were collected in RNA extraction buffer. For immunostaining, cell dissociate was fixed with 4% PFA, filtered with a 70 μm cell sieve, and blocked with 1.5% FCS in PBS for 10 min. Primary antibodies to M_1_ (M9808, Sigma), M_4_ (ab77956, Abcam), or M_3_ (GTX111637, GeneTex) were applied for 30 min at room temperature. Alexa 594 conjugated secondary antibody (Thermo Fisher) was applied for 10 min at room temperature. Flow analyses were performed using the LSRFortessa (BD bioscience) and the data analyzed using FlowJo software in collaboration with the QMRI flow cytometry core facility at the University of Edinburgh. To correct for the experiment-to-experiment signal intensity variance, each value obtained in an experiment was normalized by the average value obtained from all cells in that experiment. All staining and analysis were performed blind to genotype.

#### Immunoblotting

Hippocampal slices were homogenized in ice-cold homogenization buffer (10 mM HEPES pH 7.4, 2 mM EDTA, 2 mM EGTA, 1% Triton X-100, protease inhibitors and phosphatase inhibitors). Samples were boiled in Laemmli sample buffer and resolved on SDS-PAGE gels before being transferred to nitrocellulose and stained for total protein using the Memcode Reversible staining kit (Pierce). Membranes were blocked with 5% BSA in TBS + 0.1% Tween-20 for 1h, then incubated in primary antibody overnight at 4°C (M_4_ 1:500 ab77956, Abcam; M_1_ 1:1000 M9808, Sigma; M_3_ 1:1000 GTX111637, Genetex). Membranes were then incubated with HRP-conjugated secondary antibodies for 30 min (Cell Signaling), developed with Clarity ECL (BioRad), and exposed to film. Densitometry was performed on scanned blot films and quantified using ImageStudio Lite (Li-Cor). Densitometry data was normalized to total protein, which was quantified using scanned images of total protein staining and quantified using FIJI. To correct for blot-to-blot variance, each signal was normalized to the average signal of all lanes on the same blot. All gels were loaded and analyzed blind to genotype and treatment.

#### Metabolic Labeling

Hippocampal slices were prepared and recovered as above, then incubated in ACSF containing 25 μM Actinomycin D (Tocris) plus either vehicle (0.002% DMSO in ddH_2_O) or 75 nM pirenzepine (Tocris), 0.5 μM PD102807, 1 μM PD102807 (Tocris) or 5 μM VU0152100 (Sigma) for 30 min. Slices were then transferred to fresh ACSF containing 10 μCi/ml ^35^S-Met/Cys (Perkin Elmer) with vehicle or drugs as listed above for another 30 min. After labeling, slices were homogenized in ice-cold buffer (10 mM HEPES pH 7.4, 2 mM EDTA, 2 mM EGTA, 1% Triton X-100, protease inhibitors and phosphatase inhibitors). To precipitate proteins, homogenates were incubated in trichloroacetic acid (TCA: 10% final) for 10 min on ice before being centrifuged at 16,000 rpm for 10 min. The pellet was washed in ice-cold ddH_2_O and re-suspended in 1 N NaOH until dissolved, and the pH was re-adjusted to neutral using 0.33 N HCl. Triplicates of each sample were added to scintillation cocktail and read with a scintillation counter. Protein concentration of each sample was measured using BioRad DC (BioRad). Averaged triplicate counts per minute (CPM) values were divided by protein concentrations, resulting in CPM per μg protein. To control for daily variation in incorporation rate, the values obtained on each day were normalized to the ^35^S-Met/Cys ACSF used for incubation, and the average incorporation of all slices analyzed in that experiment. For autoradiography, slice homogenates were resolved on SDS-PAGE gels, transferred to nitrocellulose and exposed to a phosporimaging screen (GE Healthcare). Phosphorimages were acquired using a Typhoon scanner (GE Healthcare) and compared to total protein staining of the same membrane.

#### cAMP Concentration

Hippocampal slices were prepared and recovered as above, then incubated in ACSF containing 5 μM VU0152100 or vehicle (0.01% DMSO) for 1 hr. Slices were then transferred to fresh ACSF containing 50 μM forskolin (Sigma), 5 μM VU0152100 + 50 μM forskolin, or vehicle (0.01% DMSO) for 30 min. After stimulations slices were frozen on dry ice and immediately homogenized in ice-cold homogenization buffer (HBSS (Thermo Fisher, 14175053), 1% Triton X-100, 0.5 mM IBMX (Sigma)). Samples were centrifuged at 16,000 rpm for 5 min and 5 μL of supernatant was used to measure cAMP concentrations following the manufacturer’s instructions (CisBio, 62AM4PEB). Protein concentration of each sample was measured using BioRad DC (BioRad). Averaged triplicate cAMP concentrations (nM) were divided by protein concentrations, resulting in nM cAMP per μg protein.

#### Electrophysiology

Horizontal hippocampal slices (400 μM) were prepared from *Fmr1*^*-/y*^ and WT littermates (P25-32) in ice-cold dissection buffer (86 mM NaCl, 25 mM KCl, 1.2 mM NaH_2_PO_4_, 25 mM NaHCO_3_, mM 20 glucose, 0.5 mM CaCl_2_, 7 mM MgCl_2_, saturated with 95% O_2_ and 5% CO_2_) and an incision made through CA3. Slices were recovered for at least 2 hr at 30°C in ACSF (124 mM NaCl, 5 mM KCl, 1.25 mM NaH_2_PO_4_, 26 mM NaHCO_3_, 10 mM glucose, 2 mM CaCl_2_; 1 mM MgCl_2_, saturated with 95% O_2_ and 5% CO_2_) before being transferred to a submersion chamber heated to 30°C and perfused with ACSF containing either DMSO vehicle or VU0152100 (5 μM). Field excitatory postsynaptic potentials (fEPSP) were evoked by applying a current pulse to the Schaffer collateral pathway every 30 s with a bipolar stimulating electrode and recording with an extracellular electrode (1-3 MΩ) in stratum radiatum of hippocampal CA1. Following a 20 min stable baseline, LTD was induced by the application of S-DHPG (50 μM; 5 min) in the presence of either vehicle (0.002% DMSO in ddH_2_O) or VU0152100 (5 μM), which was present for the duration of the recording (55 min post DHPG washout). The magnitude of LTD was calculated from average fEPSP slope during the last 10 min of recording relative to fEPSP slope during the 20 min baseline.

#### AGS

Experiments were performed essentially as previously described (Osterweil et al., 2013). Naive male P23-25 mice were weighed and injected intraperitoneally (i.p.) with 56 mg/kg VU0152100 or vehicle (10% DMSO + 10% Tween-80 in PBS) and transferred to a quiet (< 60 dB ambient sound) room for 1 hr. Mice were then transferred to a transparent plastic test chamber and, after 1 min of habituation, exposed to a stimulus of > 120 dB (recorded sampling of a modified personal alarm, Radioshack model 49-1010) for 2 min. Each testing session contained mice from both genotype and treatment groups, tested with the experimenter blind to genotype and treatment. For each group, incidence of the following stages of AGS was calculated: wild running (WR; pronounced, undirected running and thrashing), clonic seizure (violent spasms accompanied by loss of balance), or tonic seizure (postural rigidity in limbs). Any animal that reached tonic seizure was immediately humanely euthanized.

### Quantification and Statistical Analysis

For qPCR, biochemistry and electrophysiology experiments, outliers > 2 SD from the mean were removed and significance (p < 0.05) was determined by repeated-measures two-way ANOVA using GraphPad Prism software. If significant effects were found by ANOVA, post hoc analyses were performed to compare individual groups using two-tailed paired or unpaired t test with Bonferroni correction for multiple comparisons. For AGS experiments significance was determined by Fisher’s Exact Test. Detailed results of all statistical analyses can be found in the figure legends.

### Data and Software Availability

The accession number for the RNA sequencing reported in this paper is GEO: GSE101823.

## Author Contributions

E.K.O., S.R.T., and S.S.S. conceived and designed the study. S.R.T. performed TRAP, qPCR, cAMP assays, and immunoblotting. S.S.S. performed FACS, qPCR, and bioinformatics. S.A.B. performed metabolic labeling and electrophysiology. S.R.L. performed metabolic labeling, slice stimulation, and immunoblotting. M.M. performed AGS experiments. O.D. assisted with bioinformatics. C.K. performed immunoblotting. E.K.O., S.R.T., and S.S.S. prepared the manuscript. P.C.K., D.J.A.W., and G.E.H. provided helpful advice and edited the manuscript.

## Supplemental Information

Document S1. Figures S1–S7 and Tables S1–S6

Document S2. Article plus Supplemental Information
