# Supplementary material for: Cell-Type-Specific Translation Profiling Reveals a Novel Strategy for Treating Fragile X Syndrome
Source: Neuron. 2017 Aug 2;95(3):550–563.e5. doi: 10.1016/j.neuron.2017.07.013 (PMC5548955; doi:10.1016/j.neuron.2017.07.013)
Supplement: Document S1. Figures S1–S7 and Tables S1–S6 [file mmc1.pdf]

Neuron, Volume *95*

Supplemental Information

Cell-Type-Specific Translation Profiling Reveals a Novel Strategy for Treating Fragile X Syndrome

Sophie R. Thomson, Sang S. Seo, Stephanie A. Barnes, Susana R. Louros, Melania Muscas, Owen Dando, Caoimhe Kirby, David J.A. Wyllie, Giles E. Hardingham, Peter C. Kind, and Emily K. Osterweil

Inventory of Supplemental Information:

1.) Figure S1 is related to Figure 1D, and shows comparison of genes in CA1-TRAP versus TRAP isolated genes from 2 different cerebellar cell types. GO analysis of differentially expressed genes shows significant enrichment of CA1 cell markers.

2.) Figure S2 is related to Figure 2A-D, and shows a comparison between the cumulative distribution of FMRP targets in the differentially expressed population (CA1-TRAP and Input) versus 5 randomly generated sets of genes of the same size.

3.) Figure S3 is related to Figure 3A, and shows that although the increase in *Chrm5* in the *Fmr1*$^{-/y}$ TRAP is valid, the expression of *Chrm5* in the hippocampus is close to background levels.

4.) Figure S4 is related to Figure 4A, and shows the enrichment of *Arc* in CA1-TRAP IPs isolated from DHPG-stimulated hippocampal slices.

5.) Figure S5 shows the unprocessed M_4 immunoblots and memcode staining for Figures 4D and 4G.

6.) Figure S6 is related to Figure 5, and shows a reduction in protein synthesis rates in both WT and *Fmr1*$^{-/y}$ hippcampal slices after M_1 antagonism by pirenzepine.

7.) Figure S7 is related to Figure 5C, and shows the effect of VU0152100 on forskolin-stimulated cAMP in *Fmr1*$^{-/y}$ hippocampal slices.

8.) Table S1 is related to the data shown in Figure 1F and 2F. The table lists genes significantly upregulated in the *Fmr1*$^{-/y}$ TRAP.

9.) Table S2 is related to the data shown in Figure 1F and 2F. The table lists genes significantly downregulated in the *Fmr1*$^{-/y}$ TRAP.

10.) Table S3 is related to the data shown in Figure 2E. The table lists protein clans significantly overlapping between *Fmr1*$^{-/y}$ TRAP enriched genes and FMRP targets.

11.) Tables S4-S6 are related to the data shown in Figure 2F. These tables list GO categories enriched in the genes differentially expressed in the *Fmr1*$^{-/y}$ CA1-TRAP.

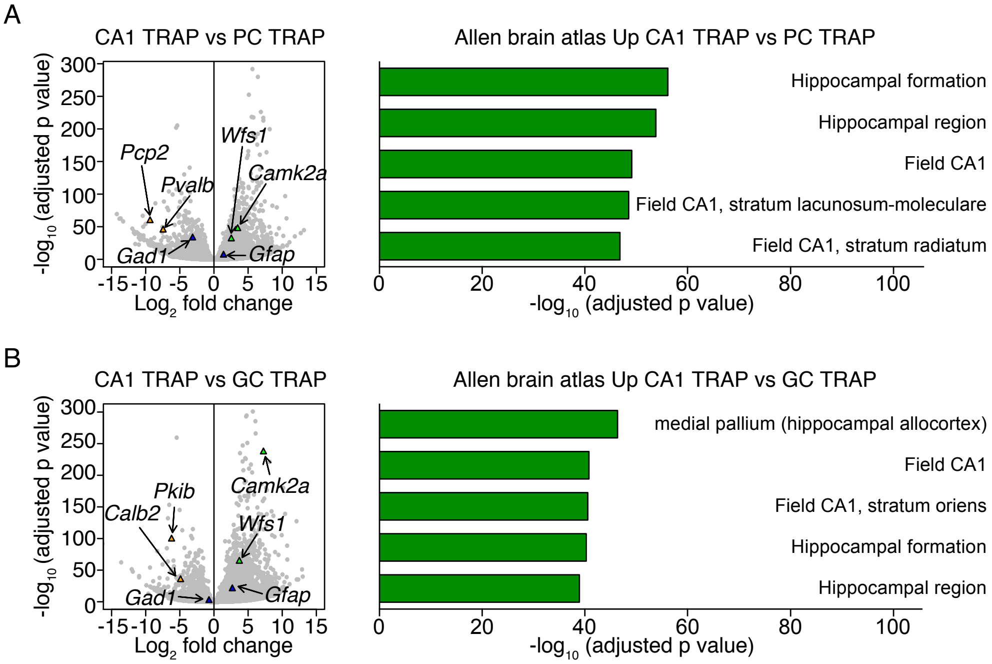

Figure S1. CA1-TRAP enriches for CA1 marker genes, related to Figure 1D. Analyses were performed to compare differentially expressed genes in CA1-TRAP versus other published cell types (Mellen et al., 2012). Comparisons between cerebellar Purkinje cells (PC) **(A)** and cerebellar granule cells (GC) **(B)** show differential enrichment of CA1 specific markers. Markers of other cell types shows little or no differential regulation. GO analyses performed on differentially upregulated genes in CA1-TRAP reveal significant enrichment of genes specific to the CA1 hippocampal region (Allen brain atlas up, EnrichR) (Chen et al., 2013).

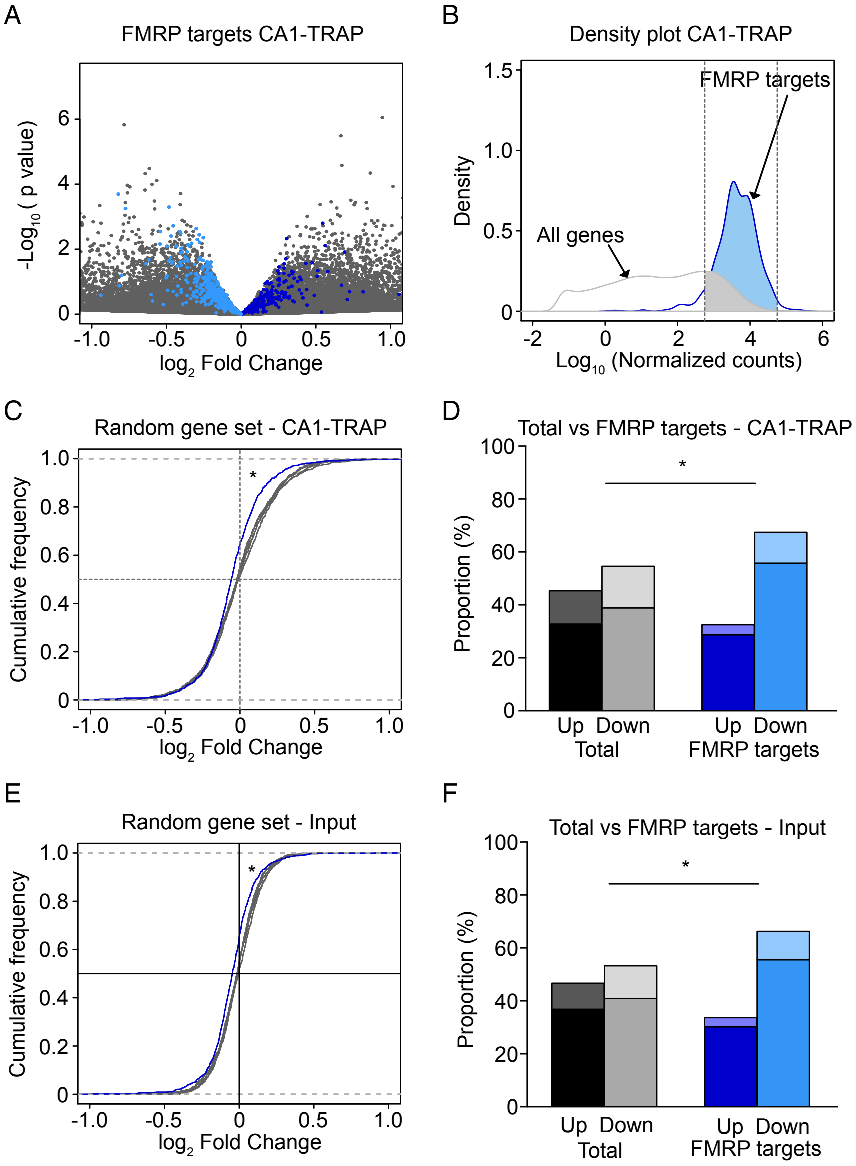

Figure S2. FMRP targets are reduced in the *Fmr1*$^{-/y}$ mRNA population, related to Figure 2A-D. (A) A DESeq2 plot of differentially expressed genes in *Fmr1*$^{-/y}$ CA1-TRAP fractions shows that more FMRP targets (blue) are decreased relative to WT rather than increased. **(B)** FMRP targets (shaded blue) and genes with a similar level of abundance (shaded gray; DESeq2 normalized counts between $1\text{x}10^{2.75}$ and $1\text{x}10^{4.75}$) were selected for downstream analysis. **(C)** A cumulative distribution of the differential expression ($\log_2$ fold change) of FMRP target genes shows a significant shift from the differential expression of 5 randomly generated sets of genes of the same size (K-S test $^*p < 1.72\text{x}10^{-7}$ (largest p-value, Benjamini and Hochberg adjusted for 5 comparisons)). **(D)** The proportion of FMRP targets downregulated in the *Fmr1*$^{-/y}$ is significantly larger than the total gene population with the same level of abundance (Fisher's exact test $^*p = 1.03\text{x}10^{-11}$). The majority of these changes are small, with a $\log_2$ fold change of less than 0.2 (dark shading), however the same pattern is seen in genes changed to a greater degree ($\log_2$ fold change > 0.2; shaded light). **(E-F)** A comparison of FMRP target versus 5 random gene sets in the Input fraction shows the same difference in cumulative distribution (K-S test $^*p < 4.19\text{x}10^{-4}$, Benjamini and Hochberg adjusted) and proportional downregulation (Fisher's exact test $^*p = 7.28\text{x}10^{-11}$).

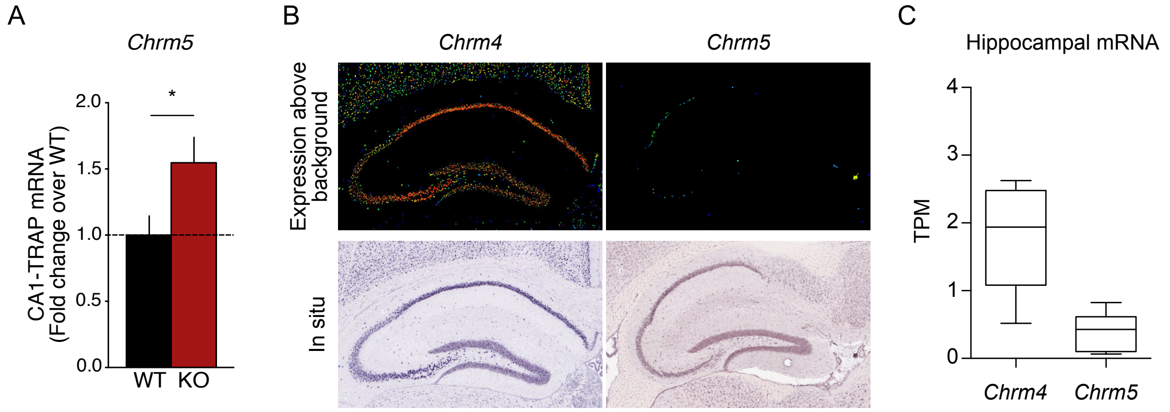

Figure S3. ***Chrm5*** **is over-translated in** ***Fmr1$^{-/y}$*** **but not highly expressed in hippocampus, related to Figure 3A.** **(A)** Follow up qPCR experiments reveal that *Chrm5* is significantly elevated in the *Fmr1$^{-/y}$* versus WT CA1-TRAP (WT = 1.00 ± 0.145, KO = 1.547 ± 0.1917, *p = 0.041, n = 17) **(B)** However, mRNA expression data from the Allen Brain Atlas (http://brain-map.org/) reveals that *Chrm4*, but not *Chrm5*, is expressed above background in the hippocampus. **(C)** Quantification of transcripts per million (TPM) in hippocampal input (from WT) shows a much lower expression level of *Chrm5* versus *Chrm4*.

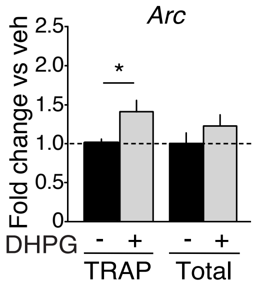

Figure S4: TRAP reveals increased translation of Arc in DHPG stimulated CA1 neurons, related to Figure 4A. Hippocampal slices were prepared and stimulated for 5 min with 50 μM S-DHPG using an mGluR-LTD induction protocol. After being removed to fresh ACSF for 25 min, slices were homogenized and processed for TRAP. A qPCR analysis of *Arc* mRNA reveals that DHPG increases expression of *Arc* mRNA in CA1-TRAP IP fraction (Veh = 1.00 ± 0.07, DHPG = 1.33 ± 0.06, *p = 0.016, n = 14), with no significant increase in total mRNA (Veh = 1.00 ± 0.13, DHPG = 1.22 ± 0.14, p = 0.102, n = 11). N = number of animals. Error bars = SEM.

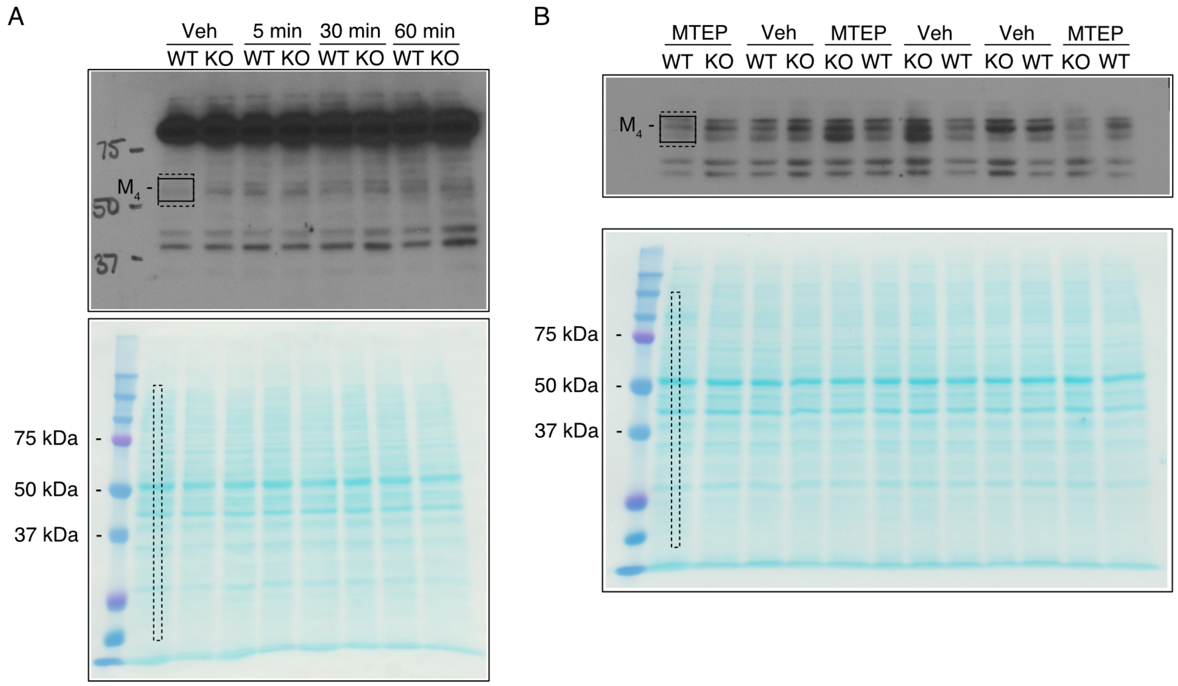

Figure S5: Unprocessed immunoblots for M_4 and corresponding membranes stained for total protein, related to Figures 4D and 4G. 10 µg of hippocampal lysate protein from WT and *Fmr1*$^{-/y}$ littermates that had been treated with DHPG **(A)** or MTEP **(B)** were resolved on the same gel, with the experimenter blind to genotype and treatment. The order in which the samples were loaded onto each gel was randomised. Immunoblotting was performed on either full blots (A) or blot strips cut between 75 KDa and 37 KDa (B; remaining strips from the same membrane were used to probe for antigens at other molecular weights). Densitometry quantification was carried out using Image Studio Lite software (Licor). M_4 bands visible at approximately 56 kDa were quantified and the average background above and below the band was subtracted (solid and dotted boxes on example immunoblot, respectively). Densitometry values were normalized to total protein of that lane. Total protein was visualized using Pierce Memcode Reversible staining kit and quantified using FIJI (dotted box on total protein staining).

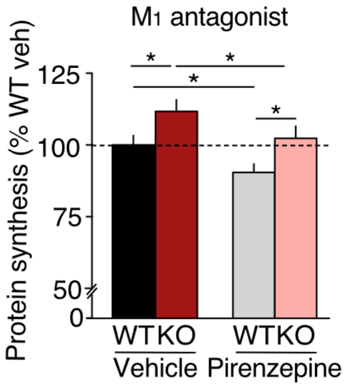

Figure S6: Antagonism of M_1 by pirenzepine results in a reduction of protein synthesis in both WT and *Fmr1$^{-/y}$* hippocampal slices, related to Figure 5. Treatment with M_1 antagonist pirenzepine (75 nM) significantly reduces protein synthesis in both WT and *Fmr1$^{-/y}$* slices (WT veh = 100 ± 3.03%, KO veh = 112.8 ± 3.53%, WT Pz = 89.44 ± 2.71%, KO Pz = 102.5 ± 3.69%, ANOVA genotype *p = 0.018, treatment *p = 0.011, WT veh v Pz *p = 0.021, KO veh v Pz *p = 0.048, n =15). N = number of littermate pairs. Error bars = SEM.

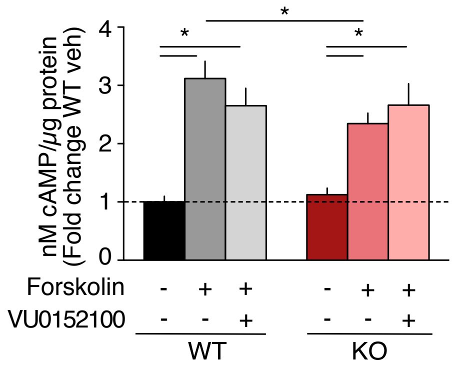

Figure S7: Effect of VU0152100 on forskolin-stimulated cAMP in *Fmr1*$^{-/y}$ hippocampal slices, related to Figure 5C. Slices were incubated with vehicle or 5 μM VU0152100 for then treated with 50 μM forskolin or vehicle for 30 minutes. As expected, forskolin stimulation significantly increased cAMP concentration in both genotypes, but to a lesser extent in the *Fmr1*$^{-/y}$ (WT veh = 1.00 ± 0.09, WT FSK = 3.11 ± 0.29, KO veh = 1.12 ± 0.110, KO FSK = 2.34 ± 0.179, ANOVA treatment *p < 0.0001, genotype x treatment *p = 0.0264, WT FSK v KO FSK *p = 0.0214, n = 9). VU0152100 pre-treatment did not result in a significant change in cAMP stimulation in either WT or *Fmr1*$^{-/y}$ hippocampal slices (WT VU = 2.879 ± 0.295, KO VU = 2.882 ± 0.357, WT FSK v WT VU p < 0.306, KO FSK v KO VU p < 0.4625, n = 9). We therefore conclude that VU0152100 does not exert its effect on protein synthesis in *Fmr1*$^{-/y}$ through cAMP signalling. N = number of littermate pairs. Error bars = SEM.

Table S1: Identified genes significantly upregulated in *Fmr1$^{-/y}$* CA1-TRAP, related to the data shown in Figure 1F and 2F.

Gene	Log_2 fold change	p value	Adjusted p value (FDR)
Ribc1	5.075	3.78E-08	0.001
Fbxw9	2.511	1.81E-07	0.002
Efnb2	0.945	8.95E-07	0.003
Kifc1	6.805	1.92E-06	0.004
Ptgds	0.668	3.25E-06	0.007
Ap5b1	2.896	1.56E-05	0.021
Cdh23	4.427	4.63E-05	0.028
Chrm5	**2.660**	**4.39E-05**	**0.028**
Csf2rb	5.186	4.50E-05	0.028
Dffa	0.674	2.65E-05	0.028
Dnase1	3.744	3.69E-05	0.028
Dvl2	1.962	3.42E-05	0.028
Fastk	0.865	4.56E-05	0.028
Fndc8	5.528	3.82E-05	0.028
Plch1	1.282	4.22E-05	0.028
Igf2os	8.980	7.90E-05	0.038
L3mbtl4	8.302	8.10E-05	0.038
Sh2d4b	5.048	9.41E-05	0.043
Stard3nl	1.016	1.18E-04	0.048
Acer2	2.610	1.35E-04	0.050
Dph1	3.316	1.41E-04	0.051
Slc35f2	3.398	1.49E-04	0.051
Gale	2.817	1.65E-04	0.054
Mettl17	1.400	2.06E-04	0.059
Ece2	1.084	2.64E-04	0.069
Cfap43	2.255	3.08E-04	0.074
Psmd3	0.734	3.14E-04	0.074
Teddm2	1.952	2.97E-04	0.074
Crhbp	1.171	3.30E-04	0.075
Cript	0.603	3.51E-04	0.078
Hs6st2	0.838	3.66E-04	0.079
Rnd2	0.765	3.64E-04	0.079
Ankub1	3.175	4.40E-04	0.085
Fgf13	0.528	4.25E-04	0.085
Gpatch3	3.335	4.19E-04	0.085
Mir453	4.910	4.53E-04	0.085
Nectin4	2.423	4.38E-04	0.085
Ppil2	0.756	4.31E-04	0.085
Selenbp2	7.745	4.12E-04	0.085
Myl4	3.560	4.72E-04	0.086
Acot12	4.682	4.98E-04	0.089
Nat8f5	4.502	5.08E-04	0.089
Anapc13	1.354	5.52E-04	0.090
Eif2ak2	1.231	5.56E-04	0.090
Kctd13	0.639	5.42E-04	0.090
Wdr81	0.843	5.65E-04	0.090
Chrm4	**1.825**	**6.41E-04**	**0.099**
Pecr	3.550	6.48E-04	0.099

Table S2: Identified genes significantly downregulated in *Fmr1*$^{-/y}$ CA1-TRAP, related to the data shown in Figure 1F and 2F.

Gene name	Log_2 fold change	p value	Adjusted p value (FDR)
Fmr1	-1.651	2.69E-07	0.002
Cep68	-0.783	1.49E-06	0.004
Vit	-2.968	7.13E-06	0.012
Csad	-2.042	2.82E-05	0.028
Mphosph9	-0.642	4.74E-05	0.028
Parg	-0.614	3.29E-05	0.028
Pkmyt1	-4.171	2.78E-05	0.028
Dusp4	-1.501	5.34E-05	0.030
Plekhh1	-0.565	7.73E-05	0.038
Pls3	-0.597	1.02E-04	0.044
Mag	-0.762	1.09E-04	0.046
Ikzf4	-0.745	1.21E-04	0.048
AI607873	-8.980	1.27E-04	0.049
Clec5a	-3.200	1.46E-04	0.051
Sox9	-0.643	1.52E-04	0.051
Sall4	-5.330	1.65E-04	0.054
AW822252	-2.854	1.78E-04	0.056
Gprin3	-0.761	1.86E-04	0.057
Usp33	-0.406	1.96E-04	0.058
Cacna1d	**-0.822**	**2.02E-04**	**0.059**
Ccdc114	-4.613	2.76E-04	0.069
Mpeg1	-1.154	2.54E-04	0.069
Cdca4	-2.137	3.03E-04	0.074
Rbpms2	-2.942	3.13E-04	0.074
Trip13	-3.749	3.34E-04	0.075
Wipf1	-0.775	3.72E-04	0.079
Ankrd44	-0.947	4.56E-04	0.085
Cdc73	-0.407	4.29E-04	0.085
Hells	-1.906	4.54E-04	0.085
Arhgef17	**-0.482**	**5.10E-04**	**0.089**
Glb1	-1.566	5.24E-04	0.089
Gpr165	-1.867	5.54E-04	0.090
Pcdhgc5	**-0.774**	**5.62E-04**	**0.090**
Ugt8a	-0.579	5.79E-04	0.092
Hdhd3	-2.614	6.37E-04	0.099

Table S3: Functional categorization of differentially expressed genes in *Fmr1*$^{-/y}$ TRAP versus FMRP targets, related to the data shown in Figure 2E.

Overlap	Protein clan	Description	DE genes	FMRP target genes
Overlapping	CL0011	Immunoglobulin superfamily	*Nectin4, Mag*	*Ncan, Bcan, Ptprs, Trio, Unc5a, Speg, Lrrm2, Ptprd, Dscaml1, Ptprf, Igsf9b, Sirpa, Ncam1, Lrrc4b, Lrrc4, Dscam, Lrr4c, Ntrk2, Ntrk3, Kalrn, Opcml*
	CL0023	P-loop containing nucleoside triphosphate hydrolase superfamily	*Rnd2, Trip13, Hells, Cdc73, Hs6st2, Kifc1*	*Myo18a, Pex6, Cacnb3, Kifc2, Chd5, Atp6v1b2, Cnp, Kif1a, Eef1a2, Cacnb4, Chd3, Dync1h1, Nav3, Kif3c, Kif1c, Dlg4, Myh10, Rhobtb2, Myo10, Kif21a, Abca3, Gnal, Smarca2, Atp5b, Kif5c, Dnm1, Abca2, Gnas, Ep400, Tjp1, Atrx, Gnao1, Smarca4, Dhx30, Rab6b, Chst2, Nsf, Myo5a, Eef2, Arhgap5, Opa1, Myo16, Znfx1, Dicer1, Kif21b, Ddx24, Dhx9, Setx, Dync2h1, Diras2 ,Nwd1 ,Arf3 ,Dlg2, Chd8, Ndst1, Rhob, Chd6, Kif1b, Chd4, Kif5a*
	CL0159	Ig-like fold superfamily (E-set)	*Cdh23, Fndc8, Csf2rb, Pcdhgc5*	*Ap2a2, Ap1b1, Ptprs, Camta1, Trim9, Ptprg, Pcdhga8, Celsr3, Ptprj, Epha4, Plxna2, Trim2, Ptprd, Pcdh7, Rimbp2, Plxna4, Plxna1, Plxnd1, Dscaml1, Pkd1, Mycbp2, Ptprf, Igsf9b, Ap2b1, Pcdh17, Trim3, Ncam1, Clstn1, Camta2, Fat4, Pcdh10, Sorl1, Dscam, Ptprt, Plxnb1, Fat2, Pcdh9, Ap2a1, Kalrn, Celsr2, Ptprz1, Fat3, Pcdhga10, Pcdhga12, Pcdhga9, Pcdhgc5, Pcdhac2, Pcdhga11, Pcdhgb2, Pcdhgc3, Pcdhgb1, Pcdhgb6,*

				Pcdhga1, Pcdhga2, Pcdhga7, Pcdhga5, Pcdhgb4, Pcdhga4, Pcdhgb5, Pcdhga6, Pcdhgb7, Pcdha4, Pcdhga3
	CL0220	EF-hand like superfamily	*Pls3, Plch1, Myl4*	*Usp32, Calm1, Camta1, Calm3, Myh10, Tppp, Myo10, Itsn1, Scn8a, Dtna, Plce1, Dst, Rasgrp1, Eps15, Macf1, Plch2, Sparcl1, Ube3b, Myo5a, Plcl2, Camta2, Slc25a23, Nrgn*
	CL0361	Classical C2H2 and C2H2 zinc fingers	*Ikzf4, Sall4*	*Peg3, Hivep2, Hivep1, Zfr, Zfp521, Zfp142, Zeb2, Hivep3, Maz, Pde4dip, Egr1, Zbtb38, Zfhx2, Zfp536, Zfp423, Tshz1, Atmin, Sall2, Atxn7l3, Zfp462, Zfp827*
	CL0465	Ankyrin repeat superfamily	*Ankrd44, Ankub1*	*Git1, Ehmt2, Clip3, Ppp1r13b, Dapk1, Shank3, Mib1, Tnks, Ank1, Fem1b, Ank2, Caskin1, Ankrd12, Ankrd11, Ehmt1, Shank2, Shank1, Myo16, Ankib1, Tanc2, Ankrd17, Anks1b, Ank3*
Non-overlapping	CL0192	Family A G protein-coupled receptor like superfamily	*Gpr165, Chrm4, Chrm5*	*Celsr3, Gabbr1, Gpr162, Gabbr2, Cnr1, Gpr158, Grm5, Grm4, Celsr2*
	CL0384	PLC-like phosphodiesterases	*Plch1*	*Plce1, Plch2, Plcl2*

Table S4: Top 5 most significant GO enrichment categories for genes differentially expressed in *Fmr1*$^{-/y}$ TRAP, related to the data shown in Figure 2F.

Go term	Overlap	P value	Z score	Combined score	Genes
Acetylcholine receptor activity (GO:0015464)	2/18	0.002505675	-3.17	5.14	*Chrm4, Chrm5*
Microtubule binding (GO:0008017)	4/171	0.004110536	-2.40	3.89	*Kifc1, Fmr1, Cript, Fgf13*
bHLH transcription factor binding (GO:0043425)	2/27	0.00524048	-2.42	3.91	*Sox9, Ikzf4*
Phosphatidylinositol phospholipase C activity (GO:0004435)	2/28	0.005601436	-2.71	4.39	*Chrm5, Plch1*
Phospholipase C activity (GO:0004629)	2/30	0.006356568	-2.64	4.27	*Chrm5, Plch1*

Table S5: Top 5 most significant GO enrichment categories for genes upregulated in *Fmr1*$^{-/y}$ TRAP, related to the data shown in Figure 2F.

Go term	Overlap	P value	Z score	Combined score	Genes
G-protein coupled acetylcholine receptor signaling pathway (GO:0007213)	2/12	0.000217241	-2.78	6.81	*Chrm4, Chrm5*
regulation of collateral sprouting (GO:0048670)	2/14	0.000298832	-2.48	6.07	*Fgf13, Rnd2*
cellular response to drug (GO:0035690)	2/49	0.003707688	-2.26	3.31	*Acer2, Crhbp*
protein polyubiquitination (GO:0000209)	3/186	0.004874445	-2.25	3.29	*Anapc13, Ppil2, Psmd3*
adenylate cyclase-inhibiting G-protein coupled receptor signaling pathway (GO:0007193)	2/62	0.005872656	-2.11	3.09	*Chrm4, Chrm5*

Table S6: Top 5 most significant GO enrichment categories for genes downregulated in *Fmr1*$^{-/y}$ TRAP, related to the data shown in Figure 2F.

Go term	Overlap	P value	Z score	Combined score	Genes
stem cell maintenance (GO:0019827)	3/109	0.000696115	-2.18	3.64	*Sall4, Sox9, Cdc73*
sulfur compound catabolic process (GO:0044273)	2/43	0.00214969	-2.32	3.87	*Glb1, Csad*
centrosome organization (GO:0051297)	2/64	0.004699961	-2.10	3.52	*Cep68, Usp33*
microtubule organizing center organization (GO:0031023)	2/70	0.005596655	-2.13	3.56	*Cep68, Usp33*
neurotrophin TRK receptor signaling pathway (GO:0048011)	3/274	0.009407354	-2.42	4.05	*Dusp4, Mag, Arhgef17*
